# Molecular Characterization of Auxin Efflux Carrier- ABCB1 in hexaploid wheat

**DOI:** 10.1038/s41598-019-51482-5

**Published:** 2019-11-22

**Authors:** Amita Mohan, Amandeep K. Dhaliwal, Ragupathi Nagarajan, Kulvinder S. Gill

**Affiliations:** Department of Crop and Soil Sciences, Washington State University, Pullman, WA 99164 USA

**Keywords:** Molecular biology, Plant molecular biology

## Abstract

Auxin is an important phytohormone that regulates response, differentiation, and development of plant cell, tissue, and organs. Along with its local production, long-distance transport coordinated by the efflux/influx membrane transporters is instrumental in plant development and architecture. In the present study, we cloned and characterized a wheat (*Triticum aestivum*) auxin efflux carrier ABCB1. The TaABCB1 was physically localized to the proximal 15% of the short arm of wheat homoeologous group 7 chromosomes. Size of the Chinese spring (CS) homoeologs genomic copies ranged from 5.3–6.2 kb with the *7A* copy being the largest due to novel insertions in its third intron. The three homoeologous copies share 95–97% sequence similarity at the nucleotide, 98–99% amino acid, and overall Q-score of 0.98 at 3-D structure level. Though detected in all analyzed tissues, *TaABCB1* predominantly expressed in the meristematic tissues likely due to the presence of meristem-specific activation regulatory element identified in the promoter region. RNAi plants of *TaABCB1* gene resulted in reduced plant height and increased seed width. Promoter analysis revealed several responsive elements detected in the promoter region including that for different hormones as auxin, gibberellic acid, jasmonic acid and abscisic acid, light, and circadian regulated elements.

## Introduction

The role of auxin as a growth regulator was perceived long before it was isolated from plant tissue^1–3^. In plants, indole-3-acetic acid (IAA) is the predominant form of auxin that plays indispensable roles in plant growth and development via its biosynthesis, distribution, and perception. Morpho-physiological responses governing the plant differentiation and architecture are governed by auxin concentration that is tightly regulated by its production and transport. Along with its local production, the auxin synthesized in the shoot apical meristem (SAM), leaf primordia, young leaves, and other actively dividing cells is transported through directional flow via vascular and bundle sheath cells, generating a concentration gradient. This directional flow or polar auxin transport (PAT) is mediated via auxin influx/efflux trans-membrane proteins including AUX1/LIKE AUX1 (AUX1/LAX), PIN-FORMED (PIN) and ATP-binding cassette subfamily B (ABCB)(for details see review^4,5^). Role of ABCB proteins in auxin transport is well established by its direct binding with auxin efflux inhibitor 1-naphthylphthalamic acid (NPA)^6–8^, *in*-*silico* docking with natural and synthetic auxin^9^ or experimentally either by radiolabeled IAA^10–12^ or by yeast and HeLa Cell assay^12,13^. Further, rootward reduction in auxin transport (up to 60%) was reported in ABCB mutants^7,11,14^. Mutants identified for efflux/influx carrier genes have clearly shown that PAT is critical for signaling, perception and gene regulation via auxin responsive elements resulting in morpho-physiological responses for plant development^15–17^.

ABCB1 was first identified in plants while searching for genes conferring cross-resistance to herbicides^18,19^. Its mutant allele in *Arabidopsis*, *abcb1*, produced differential height response under short and long day conditions^15,20^, while a double mutant *abcb1abcb19*, reduced height and increased secondary inflorescences^15^. In maize, mutation in *ABCB1 (brachytic2*) resulted in reduced plant height due to compact lower internodes, increased stalk strength, increased vasculature and broad upper leaves^21,22^. A similar phenotype was observed in commercially grown sorghum (*dw3*) mutant^21^. Similarly in apple, reduced expression of *ABCB1* in dwarf genotypes indicated its role in controlling tree height^23^. Since auxin has a positive impact on the growth and size of aerial parts of the plant, the differential accumulation of auxin in *abcb1* mutants enhances the size of the apical inflorescence in sorghum^24^ and tassel in maize^21^. Other than auxin, GA (Gibberellic acid) and brassinosteroid (BR) mutants were also reported to regulate the plant height.

Plant height is an important agronomic trait and its alteration via defective GA signaling has revolutionized the production of two major cereals, wheat (*rht*) and rice (*sd-1*), around the world^25,26^. Other than GA, brassinosteroid mutant *bri1* is commercially utilized in semi-dwarf winter barley varieties in Japan, the Korean peninsula and China^27,28^. While these semi-dwarf varieties provided lodging resistance and improved harvest index, there are negative effects associated with these mutations, for example, *bri1* mutant is less tolerant to salt stress^27^ and has reduced grain size^28^, *rht* mutants have reduced cell elongation and cell size^29^ thus negatively affect the early leaf size^30^, length of leaf blade, flag leaf sheath, grain weight^31^, coleoptile length, vigor, root biomass, leaf width, and seedling emergence under dryland^32–35^. In sorghum and maize, the GA mutants also exhibit defects in reproductive organs^31,36^. On the other hand, auxin transport mutants in maize and sorghum are particularly promising as these mutants reduce the plant height without any known negative effect on other plant traits^21,37^ probably because these mutants are not compromised in the biosynthesis or perception of auxin but only in their transport.

Encouraged by auxin mutants and their agronomic traits, in our earlier study, we reported the identification of the ‘true orthologs’ of maize *ABCB1* in wheat, barley, rice, *Brachypodium*, and soybean along with detailed gene structure and its evolution^38^. We also reported that in spite of the differences in gene structures and sequences, the 3-D conformation and auxin binding residues were highly conserved among the orthologs indicating its probable similar function. In the present study, we cloned and characterized the *TaABCB1*, analyzed its predicted promoter sequences, studied its spatial and temporal expression pattern, and showed its role in influencing agronomic characteristics in bread wheat.

## Results

### Cloning and sequence analysis of homoeologous copies of *TaABCB1*

Using genome specific primers, 6,245 bp fragment corresponding to *A*, 5,335 bp to *B*, and 5,356 bp to *D* genomes were PCR amplified and cloned from Chinese spring (Table 1 and Supplementary Figs S1 and S2). For cDNA cloning, the *A*-specific primers amplified a 4,101 bp fragment. For each of the *B*- and *D*- cDNA copies, two pairs of primers amplified two fragments, 1,887 bp and 2,330 bp with an overlap of 110 bp for *B* and 1,890 bp and 2,348 bp with an overlap of 110 bp for *D*.

**Table 1:** Size of genomic, cDNA, and predicted amino acid sequences of three homoeologous copies of *TaABCB1*.

TaABCB1 copies	gABCB1 (bp)	cABCB1 (bp)	Predicted amino acids (aa)
A	6245	4101	1367
B	5335	4107	1369
D	5356	4128	1376

**Figure 1:**
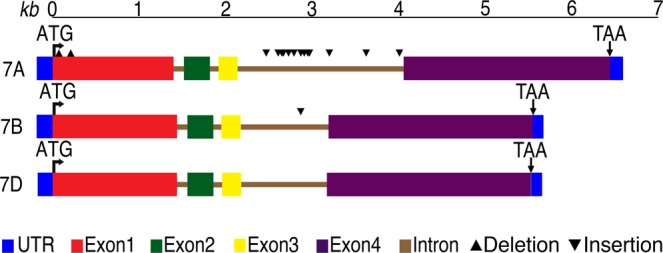
Structure of the genomic homoeologous copies (7A, 7B, and 7D) of the *TaABCB1* cloned from CS. The nucleotide sequence length was drawn to scale and the introns and exons were marked upon comparison with the cDNA sequences. Any deletions and insertions ≥10 bp in size, were marked.

At the nucleotide level, sequence coverage was 85% between *7A* and *7B*, and *7A* and *7D*, and 99.6% between *7B* and *7D* while nucleotide sequence identity ranged from 95.41–98.76% among the three homoeologous copies both at genomic as well as cDNA level (Table 2). Size of the three genomic copies varied due to deletions and insertions (Fig. 1 and Table 1), though number of exons and introns were same among homoeologs. Genomic size of *7A* was larger than *7B* and *7D* copies (Table 3) mainly due to seven insertions of 12 to 253 bp present in the third intron of *7A* copy. The *7B* copy has an insertion of 14 bp in the third intron. Exon 1 of the *7A* copy carried two insertions of 12 bp each. Additionally, insertions and deletions of < 12 bp also contributed to the gene size variation (Fig. 1).

**Table 2:** Sequence similarity among three homoeologous copies of *TaABCB1*.

TaABCB1 copies	gABCB1 (%)	cABCB1 (%)	Predicted amino acids (%)
7A–7B	95.41	98.07	98.02
7A–7D	96.71	98.76	98.32
7B–7D	95.97	98.61	98.61

**Table 3:** The size of exons and introns in homoeologous copies of *TaABCB1*. Sizes are given in base pairs (bp).

*TaABCB1* copies	Exon 1 (bp)	Intron 1 (bp)	Exon 2 (bp)	Intron 2 (bp)	Exon 3 (bp)	Intron 3 (bp)	Exon 4 (bp)
7A	1190	126	308	97	224	1921	2379
7B	1214	110	308	92	224	1026	2361
7D	1217	122	308	93	224	1013	2379

Comparing sizes, exon 3 was the smallest and exon 4 was the largest (Table 3). Sizes of exons 2 and 3 were equal among the three homoeologs whereas the other two showed some size variation. In general, size of introns was more variable than that of exons. Intron 2 was the smallest and was highly similar in size among homoeologs with a difference of 4 bp between *7A* and *7D* and 5 bp between *7A* and *7B*. Intron 1 being the largest in *7A* copy has a difference of 4 bp compared to *7D* and 16 bp from *7B*. Intron 3 was the largest and the most variable in size due to large insertions in the *7A* resulting in a difference of 908 bp from *7D* and 895 bp from *7B*.

Sequence comparison among the homoeologous copies showed that the exonic regions were relatively more conserved than the intronic regions (Table 4). Among exons, exon 3 is the most conserved with sequence similarity ranging from 99.6–100%, while among others, it ranged from 98.49–99.07%. Sequence similarity among introns ranged from 73.64 to 96.74% (Table 4) with the maximum variation for intron 1. The *7A* and *7D* copies showed higher sequence similarity compared to *7B* copy. Size similarity did not always correlate with the sequence similarity. For example, intron 2 showed only a single base pair difference between *7B* and *7D* but showed 85.87% sequence similarity. Whereas intron 3 was the most variable in size but has comparatively high sequence similarity among homoeologs (Table 4).

**Table 4:** The percent nucleotide sequence similarity for exons and introns among three homoeologous copies of *TaABCB1*.

*TaABCB1* copies	Exon 1	Intron 1	Exon 2	Intron 2	Exon 3	Intron 3	Exon 4
7A-7B	98.49	73.64	98.05	96.74	99.55	87.43	98.48
7A-7D	99.07	94.26	98.38	92.47	99.55	91.71	98.57
7B-7D	98.27	73.64	98.38	85.87	100	89.73	98.69

Comparison of homoeologs with their respective progenitors revealed evolutionary conservation. *Ae. tauschii ABCB1* showed 99.9% sequence identity and 100% coverage with the *T. aestivum 7D* copy. Similarly, *Ae. speltoides* gene showed 98.7% identity and 100% coverage with the *T. aestivum 7B* copy, and *T. urartu* gene showed 98.8% identity and 92% coverage with the *T. aestivum 7A* copy. The two insertions of 212 and 288 bps present in intron 3 of *7A* copy of hexaploid wheat were absent from *T. urartu* gene.

### Intron comparison

**Figure 2:**
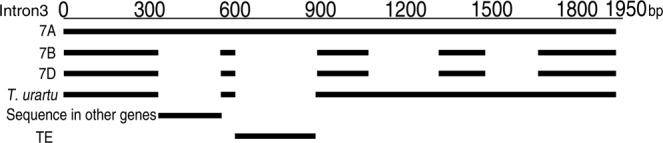
Alignment of the intron-3 of the *7A* copy with the corresponding region of the *7B*, *7D*, and the A-genome donor, *Triticum urartu*. Two *7A* specific insertions one showing sequence identity with other gene like Pm3, *F3H-A1* and *ALMT1-M77.1* and second with MITEs are also drawn below.

Compared to *7B* and *7D* copies, size of intron 3 of the *7A* copy was the largest due to the presence of four genome specific large insertions (Table 3; Fig. 2). Two of these insertions (from 1,074 bp to 1,316 bp and 1,477 bp to 1,652 bp) were also present in the ancestral diploid progenitor *T. urartu* whereas the other two insertions (from 337 bp to 548 bp and 607 bp to 888 bp) were present in the tetraploid durum ancestor cv. Strongfield. The insertion from 337 bp to 548 bp showed high sequence similarity with the wheat class II transposable elements *Tc1/mariner* (93%). This insertion was found in other wheat genic regions and showed high sequence similarity with the promoter region of various alleles of powdery mildew (*Pm3*) resistance gene (97%), gamma gliadins and LMW-A2 (97%), *F3H-A1* gene for flavanone 3-hydroxylase (95%), and exonic region of *ALMT1-M77.1* gene of *Secale cereale* (91%). The presence of the *mariner* element might influence gene expression, for example, early flowering under short day condition in cv. Mercia might be related to the *mariner-like* transposable element present in intron1^39^. The insertion at position 607 to 888 bp showed high sequence similarity with *H. vulgare* Talisker Miniature Inverted–Repeat Transposable Elements (MITEs) and was present in large number in the genomic sequences of *T. aestivum*, *H. vulgare*, and *Ae. tauschii*.

### UTR and promoter sequence comparison

**Figure 3:**
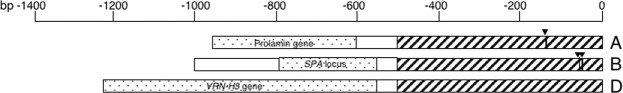
Comparison of the 5′ UTR of the three homoeologs of the *TaABCB1* gene. The 0 represent upstream of translation start site. Inverted black triangle () represent insertions. The lined box () represents high sequence similarity among the homoeologs and dotted box () represents sequence similarity with other genes sequences of VRN, Prolamine and SPA locus.

The UTRs and predicted promoter sequences were cloned from the genomic DNA of Chinese spring using gene-specific primers (see methods). Depending upon the sequence available upstream of the translation start site, 983 bp of *7A*, 1,007 bp of *7B*, and 1,217 bp of the *7D* copies were cloned (Supplementary Fig. S3A). 5′ UTR had variable size among homoeologs consisting of 152 bp in *7A*, 149 bp in *7B*, and 132 bp in *7D*. The differences were mainly due to insertions of 19 bp and 1 bp in *7A*, and 11 bp and 10 bp in the *7B* copy. The sequence similarity between 5′ UTRs of *7A* and *7B* was 83.2% compared to 94.7% between *7B* and *7D* and 96.2% between *7A* and *7D*. The sequence comparison among homoeologs revealed that 525 bp sequence present proximal to translation start site was similar and the differences were mainly present in the distal sequences. The 3′ UTR showed similar pattern of variations as of 5′ UTR, consisting of 188 bp in *7A*, 123 bp in *7B*, and 133 bp in *7D* (Supplementary Fig. S3B). The 3′ UTR of *7A* showed insertions of 10 bp and 5 bp, the *7B* has a deletion of 7 bp and an insertion of 1 bp. The *7D* copy did not show any genome specific insertion or deletion as that of 5′ UTR. Part of the upstream region of *7D* showed high sequence similarity to the promoter region of *VRN H-3*. On the other hand, corresponding sequence of the *7A* has high sequence similarity to 1DS *Prolamin* locus, and the *7B* sequence to the SPA locus (Fig. 3).

Detailed promotor analysis revealed that all homoeologs have auxin responsive motifs, *cis*-regulating light responsive elements, and elements for meristem specific activation, GA-responsiveness, endosperm expression, and for circadian control. Copy specific elements like MYB binding elements along with that for activation via ABA, jasmonate (MeJA), salicylic acid, and anoxic specific inducibility were identified in *7A* while *7B* have elements for the anaerobic induction. The *7D* have elements for low-temperature responsiveness and light responsiveness MYB binding sites. Common *cis* acting elements were present in variable numbers among homoeologs. A maximum number of copies for common *cis* acting element (CAAT-box) were identified in *7D* while core promoter element (TATA-box) was identified in the *7B*. In general, homoeolog promotors showed difference in the number of *cis*-regulatory elements with the *7A* copy carrying the maximum number (Supplementary Table S1).

### Predicted amino acid sequence analysis and ligand docking

The three homoeologs have high aa sequence similarity (Table 2). Structurally, there were differences of two to nine aa which corresponded to insertions and deletions present in the N-terminal, linker, and C-terminal regions. The N-terminal cytoplasmic region possessed deletions of five aa in 7A, two aa in 7B, and one aa in the 7D. The linker region has aa deletion of two in 7A and four in 7B. C-terminal cytoplasmic region has aa deletions of four in 7B and two in 7D.

The Conserved Domain Database (CDD) (http://www.ncbi.nlm.nih.gov/Structure/cdd/wrpsb.cgi) identified bipartite structure consisting of two trans-membrane domains (TMD) and two nucleotide-binding domains (NBD) in three homoeologs (Supplementary Fig. S4). The domains were organized as TMD1-NBD1-Linker-TMD2-NBD2, linker connecting the two domains. Each NBD domain contains nucleotide binding and hydrolysis motifs including Walker A, Q-loop, ABC transport signature, Walker B, D-loop, and H-loop motifs. NBD1 and NBD2 domains mainly differed due to sequence differences for Walker A and Q-loop motifs. Ligand binding motifs such as UM1, UM2, and UM3 and monocot specific motifs were highly conserved among homoeologs (Supplementary Fig. S4)^38^.

**Figure 4:**
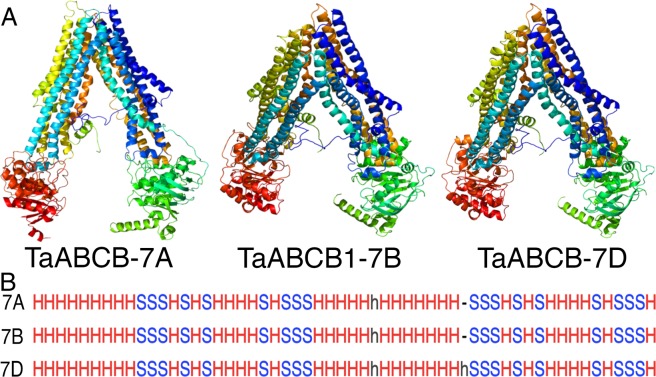
Comparison of (**A**) 3-Dimensional structures of TaABCB1 protein predicted using Phyre2; (**B**) Secondary structure alignment of TaABCB1 homeologs predicted using PDBeFold.

The *C. elegans* Phosphoglycoprotein1 (PGP1/ABCB1) homology model predicted a V-shaped inward conformation of homoeologs proteins (Fig. 4A; http://www.sbg.bio.ic.ac.uk/~phyre2/html/page.cgi?id=index). Superimposed predicted 3-D structures (http://www.ebi.ac.uk/msd-srv/ssm/cgi-bin/ssmserver) revealed an overall Q-score of 0.98, RMSD of 0.34 and 54 aligned secondary structure elements (Fig. 4B). The higher Q-score among homoeolog (Table 5) suggests that the homoeologous proteins may have similar function.

**Table 5:** 3-D structural similarity predicted using PDBeFold among the three copies of TaABCB1.

TaABCB1 Protein	N_SSE_	RMSD	Q-score
7A	55	0144	0.99
7B	55	0.22	0.99
7D	56	0.23	0.99

The predicted TaABCB1 protein has a molecular weight of 147 kD with a theoretical pI (isoelectric point) ranging from 8.11–8.26 for the three copies in agreements for membrane proteins. The aa composition revealed that the alanine is most abundant (13.7%) followed by arginine (6.6%). The instability index (II) of the predicted protein is 39.0, aliphatic index is 94 and grand average of hydropathicity (GRAVY) is 0.06 indicating the 3-D structure is thermodynamically stable and consists of helix structure.

The predicted 3-D TaABCB1 homoeologs were used for docking natural indole-3-acetic acid (IAA) molecule. The three homoeologs showed nine possible poses for each homoeolog with a binding energy ranging from −7.5 to −5.8 kcal/mol. The best IAA binding site orientation has the binding affinity of −7.4 kcal/mol for A and B homoeolog and −7.5 kcal/mol for D homoeolog with lower and upper bound RMSD of zero (Supplementary Fig. S5).

### Mapping of *TaABCB1*

**Figure 5:**
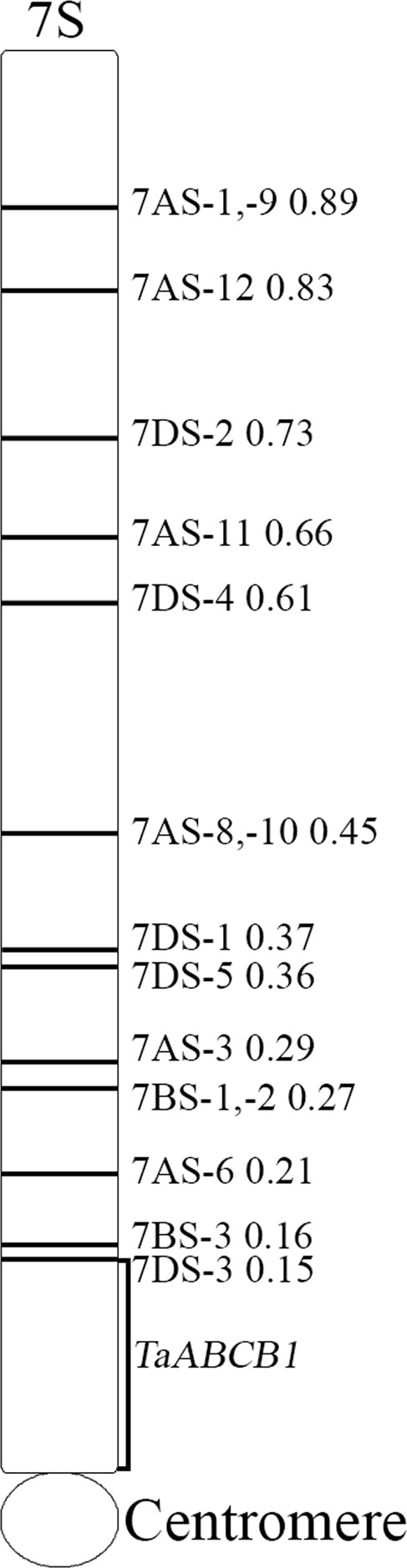
Physical mapping of the *TaABCB1* gene in wheat. The deletion breakpoints with fraction length were marked on the right of the chromosome arm and were according to Endo and Gill, 1996.

Because of significant sequence similarity among various ABC gene family members, we developed a probe specific for ABCB1 and used it for its physical mapping. The probe TaABCB1-424 detected three fragment bands in cultivar CS confirming probe’s specificity. The probe detected three bands in all NT lines except for group 7 chromosomes, short arm ditelosomic lines and deletion lines close to centromere localizing the gene on short arm of homoelogous group 7 near to centromere (Fig. 5). Localized via read Based Chromosome Assignment (http://webblast.ipk-gatersleben.de/barley/)^40^, the barley ortholog (*HvABCB1;* sequence from Dhaliwal *et al*., 2014*)* was mapped on chromosome 7HS.

In order to genetically map the *TaABCB1*, we used the composite mapping strategy. As we could not detect polymorphism with the available Synthetic x Opata mapping population in wheat, we used barley Steptoe x Morex population. TaABCB1-424 probe revealed polymorphism between Steptoe and Morex parents with *Dra*I restriction digestion. The DAK642 and ABR329 markers flanking *ABCB1* were mapped at a genetic distance of 0.1 cM on 7HS near centromere (Supplementary Fig. S6). On the basis of consensus/composite maps of both barley and wheat^41^, *TaABCB1* was placed between BCD349 and ABC455 markers of wheat.

### Expression of *TaABCB1* gene

*In*-*silico* expression of the gene was studied using the tBLASTN at the WheatExp database. The gene showed expression in all 20 tissues including root, shoot, leaf, seed, flower and floral parts. The expression was maximum in various floral tissues including stamens, pistils, spikelet and spike followed by root and stem. The expression was minimum in seed and its related tissues (Supplementary Figs S7A,B). In general, the *7B* copy showed significantly higher expression as compared to the other two copies. The expression level for the other two copies didn’t show any clear pattern as in some tissues the 7*A* copy showed higher expression whereas in other tissues the expression of *D* copy was higher.

**Figure 6:**
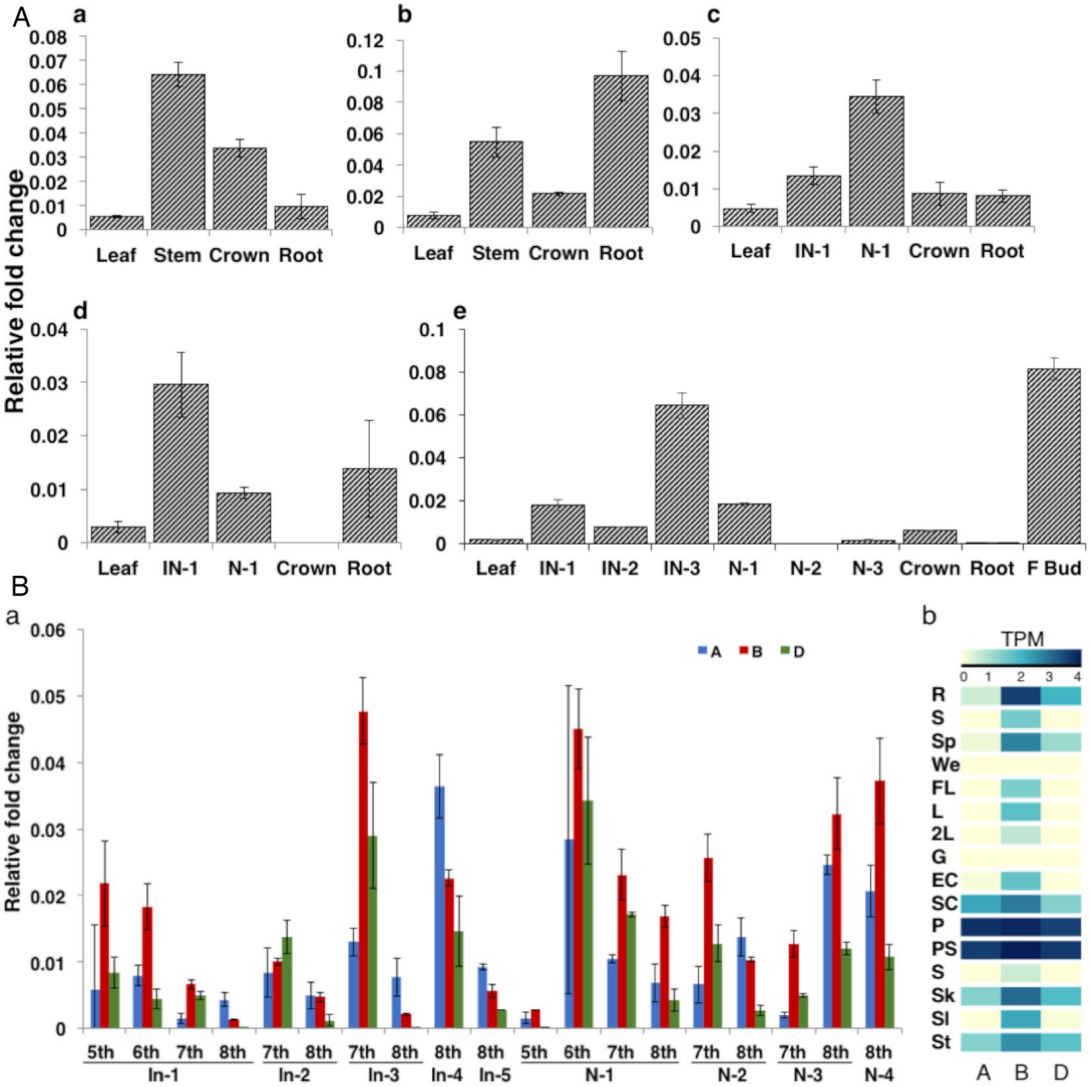
Expression pattern of *TaABCB1* gene (**A**) in various tissues at different developmental stages a. 2-week, b. 4-week, c. 5-week, d. 6-week and e. 7-week stage; (**B**) Expression of homoeolog copies of *TaABCB1* gene a. in nodes and internodes of 5, 6, 7 and 8-weeks old stem b. heat map of differential expression of homoeologs in different tissues including R-root, S-shoot, Sp-spikelet, We-whole endosperm, FL-flag leaf, L-Leaves, 2L-second leaf, G-grain, EC-endo + coat, SC-seed coat, P-pistil, PS-pistil-stamen, S-Stamen, Sk-spike, Sl-seedling, and St- stem.

Homoeolog gene expression was confirmed by SSCP analysis, as observed earlier, the gene expressed in all tissues and developmental stages. SSCP analysis on wheat NT lines identified bands corresponding to the *7A* and *7D* copies but the *7B* copy band did not resolve. The spatiotemporal expression of *7D* copy was observed with less expression in the leaves during the early development stages (Supplementary Fig. S8). qRT-PCR using common primer reveled that *TaABCB1* expressed in all tissues with relatively higher expression in stem, root and flowering bud (Fig. 6A). In stem including nodes and internodes, in general, a higher expression of *7B* than *7A* and *7D* was observed (Fig. 6B and Supplementary Fig. S7A). No clear expression pattern was observed in nodes and internodes at different development stages. Similarly, we have observed the expression variation among the different biological replications of cvs. Indian and Sariab-92 collected at the same time (Supplementary Fig. S7B), suggesting that the physiological maturity of the plant and environmental factors might influence the expression of the gene.

### *TaABCB1* characterization via Virus-induced transient gene silencing (VIGS)

**Figure 7:**
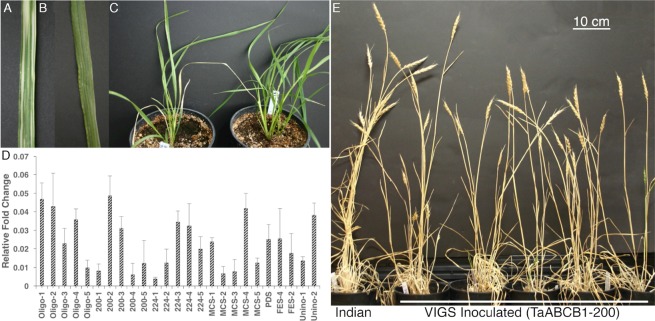
(**A**) PDS gene silencing in leaf tissue; (**B**) viral symptoms on leaf inoculated with TaABCB1; (**C**) comparison of inoculated (left) and un-inoculated (right) plants after 25 days post-inoculation; (**D**) Real Time PCR analysis showing relative expression of TaABCB1 gene in inoculated and un-inoculated plants. X-axis shows samples and Y-axis shows transcript relative to Actin control; (**E**) Plant height comparison of control (Indian) and VIGS inoculated (TaABCB1-200) plants at maturity.

In maize and sorghum, a mutation in *ABCB1* gene resulted in reduced plant height mainly due to compression of lower internodes^21^. To see if wheat ortholog also controls plant height, transient silencing of *ABCB1* using VIGS was performed by using three TaABCB1 specific sequences (material and methods). Viral symptoms and leaf photo-bleaching in the PDS inoculated plants were taken as an indicator of successful inoculations (Figs 7A,B). Compared to the uninoculated and FES inoculated plants, the MCS inoculated plants showed 1% reduction and PDS plants showed 2.4% reduction in plant height (Table 6). Both differences were non-significant however, the three oligos targeting TaABCB1 gene, average plant height reduction ranged from 14.4% to 24.1% with height ranging from 51–88 cm as compared to 75–90 cm for MCS and 72–90 cm for PDS inoculated plants (Table 6 and Fig. 7C,D). Further investigating the internode lengths, as compared to MCS, reduction in lengths was observed in all the internodes with maximum reduction in the lowest internodes. Mean percent reduction in lower internode length was 39% in TaABCB1-200, 46% in TaABCB1-224, and 34% in TaABCB1-oligo compared to MCS. The expression of gene in 21 days post inoculation leaf tissue revealed no clear pattern compared to positive inoculated and un-inoculated plants (Fig. 7E). This might be due to lower expression of *TaABCB1* in leaf tissue compared to stem and flower and further expression varied with developmental stages, physiological maturity and stage of tissue collected.

**Table 6:** Plant height (cm) of matured VIGS inoculated and un-inoculated plants at two-leaf stage.

Treatment	Height range	Average height	Average reduction relative to control
Control	75–90	83	0%
MCS	75–90	82	1%
PDS	72–90	81	2.40%
TaABCB1-200	66–88.5	71	14.40%
TaABCB1-224	60–71.5	68	18.00%
TaABCB1-oligo	51–78.5	63	24.10%

### Generation and phenotyping of *TaABCB1* RNAi plants

**Figure 8:**
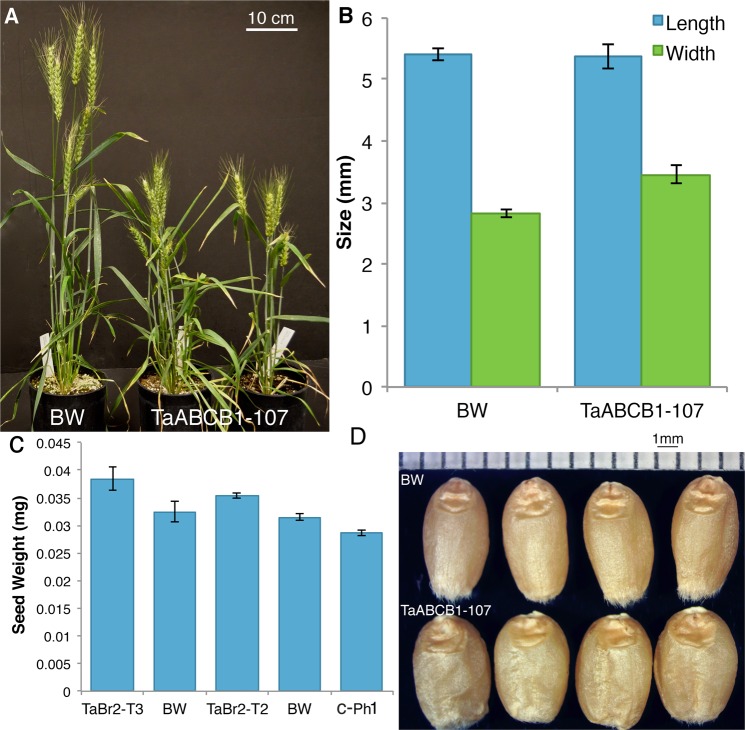
Phenotype of the TaABCB1 RNAi plant (TaABCB1-107) in comparison to the control Bobwhite (BW). (**A**) Plant height of BW and TaABCB1T2-107; (**B**) mean seed weight comparison of control BW, TaABCB1T2-107, TaABCB1T3-107 and the *C*-*Ph1* gene used as a control. BW after RNAi plants represent control grown with respective generation; (**C**) mean seed length and breadth of control and TaABCB1T3-107 mature seeds, (**D**) seed size comparisons among (**a**) Bobwhite and (b) TaABCB1T3-107.

Based on reduction in plant height with transient silencing of *TaABCB1* with VIGS, we performed the stable RNAi in cv. Bobwhite. The 108 putative transformants were screened with the primer combinations and eleven plants positive at least with three construct-specific primers were selected. Six plants each of the selected 11 selfed T1 plants along with ten Bobwhite control plants were grown in the green house to evaluate for plant height and tiller number. Of these, selected 66 plants (progeny of selected 11 plants), 25 plants showed 7.5 to 33% reduction in plant height as compared to that of the control Bobwhite. Out of 25, 9 plants had week stems and showed 35–51% reduction in tillers. All six plants of TaABCB1-107 showed reduction in plant height and thus were analyzed in detail. Compared to control, all six TaABCB1T2-107 plants showed reduction in gene expression in five weeks old flag leaf (Supplementary Fig. S9) although as reported earlier the expression of *ABCB1* was variable. Plant height of six plants ranged from 45 to 62 cm with 7 to 32% reduction relative to average height of Bobwhite control. Compared to Bobwhite, significant reduction in internodal length was observed for all internodes (Table 7 and Fig. 8A). The last internode (internode 5) was highly variable in length and was not visible in three of the six transgenic plants. Further, we have observed lower gene expression in the first and last nodes and flowering bud of 6-week old plants compared to control (data not shown). In addition to plant height, tiller number except for one of six plants compared to control were significantly higher (Table 7). The flag leaf length of the main tiller, spike length, and spikelet number of the six RNAi plants were not significantly different (at p < 0.05) from Bobwhite. There was a significant difference in the leaf width at the p < 0.05 level for the three values [F(1, 12) = 10.83, p = 0.006] (Table 8). Post Hoc comparison using the Tukey HSD test indicated that the mean leaf width for RNAi plants (M = 1.66, SD = 0.09) was significantly different than Bobwhite (M = 1.5, SD = 0.08). Compared to Bobwhite, no significant difference in coleoptile length was observed while root length was significantly shorter in the RNAi plants at p < 0.05 level for three values [F(1, 65) = 35, p = 1.37E-07)] (Table 8). Post Hoc comparison using the Tukey HSD test indicated that the mean root length for RNAi plants (M = 60.5, SE = 2.12) was significantly less than Bobwhite (M = 76, SD = 1.57). Measured at maturity, grain size and weight in RNAi plants were significantly higher compared to both Bobwhite and C-Ph1 used as transformation control (Fig. 8B). The difference in seed weight was mainly because of difference in seed width. In case of seed length, no significant differences were observed between the RNAi and Bobwhite control (Fig. 8C-D).

**Table 7:** Mean plant height, internodal (IN) length (cm) and tiller number of Bobwhite (BW) and TaABCB1-107 (T2). The data presented is of main tiller of the plant.

Character	BW		TaABCB1-107		P-value
	Mean	Range	Mean	Range	
Plant Height*	67.1	64–70	55.5	45–62	7.11E-05
IN 1 (Peduncle)*	27.27	26–29	24.5	18–27	0.019
IN 2*	14.14	13–15.5	12.17	9–14	0.009
IN 3*	8.09	7–9	5.5	5–7	5.8E-06
IN 4*	5.64	5–7	3.67	1.5–7	0.0082
IN 5^§^	—	0.5−3	—	0–2	—
Tiller number*	6.18	5–8	8.17	3–11	0.05

^§^Highly variable no mean length calculated, *ANOVA significant at p ≤ 0.05.

**Table 8:** Morphological characteristics of Bobwhite and TaABCB1-107 (T2) plants. Data on coleoptile and root length (mm) was taken at the seedling stage and data on flag leaf and spike (cm) was of the main tiller of the plant.

Character	BW		TaABCB1-107	
	Mean	Range	Mean	Range
Coleoptile length	72.46	58–85	73.09	55–88
Root length^*^	75.45	62–100	63.51	36–83
Flag leaf length	29.14	27.0–32.0	31.71	25.5–35.0
Flag leaf width^*^	1.51	1.4–1.6	1.66	1.5–1.8
Spike length	11.26	10.0–12.3	11.50	11.0–12.0
Spikelet number	19.86	18–22	21.29	20–23

^*^ANOVA significant at p ≤ 0.05

## Discussion

ABCB1, belonging to a large family of ATP-binding cassette (ABC) transporters, is functionally conserved as an efflux transporter in plants, human, mouse, and *C*. *elegans* to transport various substrates across the membrane. In plants, the ABCB1 is involved in long-distance auxin transport influencing morpho-physiological responses. Among cereals, maize *ABCB1* (*br2*) is well characterized^21^ and showed high sequence and structural conservation among monocots and dicots^38^.

The three genomes of wheat though evolved independently share similarities as observed with gene structure and sequences of *TaABCB1* homoeologs. Even though the coding DNA sequence (CDS) and protein length are quite similar among homoeologs, differences were observed in size and sequences of introns, implying that introns may have differentially evolved and accumulated changes. Some of these changes might be accumulated during polyploidization as observed in the third intron of *7A* carrying four genome specific insertions that were absent in *7B* and *7D*. Two of these insertions were present in the ancestral progenitor *T. urartu* and the remaining two insertions were probably originated during/after polyploidization, as these were absent in the ancestral diploid genome but present in tetraploid progenitor. Major reason of expansion in size is due to insertion of transposable elements like in intron 3 of *7A*. One of these insertions corresponds to a *mariner*-transposable element inserted in the genic part as was also found in promoter regions of *Pm3* and *F3H-A1* genes of wheat, *ALMT1-M77.1* gene of *Secale cereale* and in some uncharacterized sequences of chromosome 3B in *T. aestivum*. The second *7A* specific insertion corresponded to MITE also present in a number of genes across different species including uncharacterized sequences in 1DS of *T. aestivum*, Prolamin locus of *Ae. tauschii*, and *Sod1* and *Hox1* genes of *H. vulgare*. MITE sequence with a length of 285 bp in *7A* has conserved terminal inverted repeats of GAGCA and the target site duplication sequence of TTA as that of MITE Talisker in barley^42^, but surprisingly MITE sequence is absent from barley *ABCB1*. Irrespective of the changes in intron sizes, the three copies have same intron phase distribution indicating the conserved intron-exon boundaries.

Although majority of genes in wheat are present in three copies, differential expression of homoeologs is well documented^43–46^. We observed variation and differential spatiotemporal expression of *ABCB1* possibly influenced by several factors including the developmental stage, alignment with respect to plant, light and time of the day. Further, 5′ UTR plays an important role in the regulation of gene expression and some differences in expression pattern were speculated to be due to changes in the 5′ UTR^47^. This variable expression might be due to the presence of *cis*-regulatory elements. In *7A*, genome specific insertions carried additional regulatory motifs like abscisic acid responsive elements (ABRE), suggesting cross talk between auxin and ABA. Corresponding element in sorghum might have upregulated *ABCB1* expression under IAA and ABA treatment^48^. Additionally, lower expression of 7A  copy might also be explained by the presence of *mariner*-transposable element^49^. Even though upstream sequence of putative promoters of three copies showed very less similarity, auxin responsive element (GAGAC) was uniformly present indicating its role in auxin transport. The *7D* promoter showed sequence homology with promoter sequence of *VRN-H3* and *7HS* of barley suggesting its divergence from a common ancestor about 8–9 million years ago (MYA).

Although copy-specific and spatiotemporal expression was observed, homoeologs in general showed higher expression in meristematic tissues including spike, spikelets, stem, roots and crown. Similar pattern was reported for *Arabidopsis*, maize and apple *ABCB1* genes^8,19,22,23,50^. This is expected as actively dividing cells are well known to produce auxins. Presence of meristematic active elements in promoter might be responsible for its higher meristem expression. We have also observed higher expression of *ABCB1* in spikelets. Auxin concentration plays a crucial role in the development of the reproductive organs as its mutants showed disproportionate growth and malformation of the reproductive organs^51,52^.

Differences in the plant architecture is probably the reason that in wheat oppose to maize (and sorghum), we observed reduction in all the internodal lengths. In VIGS and RNAi plants, the maximum reduction occurred in the lowermost internode, that either become very small or altogether indistinguishable. Auxin promotes growth and elongation of several organs. On an average, the seeds harvested from RNAi plants were 22% wider than the Bobwhite. Although ABCB1 controlling the seed size has not been reported, though involvement of auxin transporters in regulating seed size in tomato and rice has been reported^53–55^. The difference in seed weight was primarily due to increase in seed width as no difference was observed for seed length. Seedlings of RNAi plants showed no significant differences in coleoptile length but surprisingly showed reduction in root length. Auxin has been shown to reduce root growth, thus, we expected deeper roots in the case of RNAi plants as reduced auxin transport should result in lower levels of auxin in the roots. We do not however know the level of auxin in the RNAi plants so can’t make accurate determination of the effect of auxin level on the wheat root growth. Additionally, as reported earlier we have also observed early pollen maturity and dehiscence in RNAi plants compared to control^56^.

We have observed variation in both *in-vivo* and *in*-*silico* expression of the ABCB1 and the expression was not in complete agreement with the observed phenotype in case of VIGS plants. Similarly, we observed variation in internodes and leaf of RNAi plants while expression reduction in meristematic tissue like nodes and flowering buds was consistent. This variation might be due to interplay of several factors including the physiological maturity of the plants and response to growing micro-environment conditions. These results are in agreement with the anti-sense *Arabidopsis* lines, where the protein levels were not correlated with the observed phenotypes^19^. Additionally, the presence of circadian and light regulated elements might explain variation in expression of the gene. Currently its being reported that there is a crosstalk among different hormone signal transduction pathways^57^. We have also observed several regulatory elements for different hormones in the promotor indicating the expression of the gene might be modulated by different hormones.

## Materials and Methods

### Plant material and growth conditions

Cultivar Chinese Spring (CS) was used for TaABCB1 gene cloning, Nullisomic-tetrasomic (NT) and Ditelosomic (DT) lines in CS background^58^ were used for mapping. Wheat cultivar Indian, lacking any known *rht* mutations was chosen for the Single-Strand Conformation Polymorphism (SSCP), real-time expression analysis, and Virus Induced Gene Silencing (VIGS). All plants were propagated in 6-inch pots using Sunshine#1 potting mixture (SunGro Horticulture, Bellevue, WA, USA) supplemented with 14 g Nutricote 14–14–14 fertilizer (Plantco Inc., Brampton, Ontario, Canada). Plants supplied with equal amount of soil and water, were grown in a Conviron PGR15 growth chamber equipped with high-intensity discharge lamps at 23 °C temperature and 16 hr light (500–700 μmol m^−2^ s^−1^).

### Oligo synthesis

The identified ‘true ortholog’ wheat Expressed Sequence Tags (ESTs) - CA730599 and CJ800530^38^ were aligned with ZmABCB1 using “Align X” module of the Vector NTI Advance™ 11.0. The unique regions (brown line in Supplementary Fig. S10), with high sequence similarity with ZmABCB1 but devoid of any motifs/domains, synthesized in-frame together as a TaABCB1-oligo (TaABCB1-200; 200 bp and TaABCB1-224; 224 bp; Supplementary Fig. S11 and Supplementary Table S2) was cloned in a pUC59 vector (Genscript Inc, USA). The fragments TaABCB1-200 and TaABCB1-224 released by *Not*I-*Pac*I digestion were cloned in BSMV gamma (pγ.bPDS4) for VIGS. The fragment TaABCB1-424 released by *Not*I digestion was used for RNAi and as a probe for physical and genetic mapping.

### Physical and genetic mapping of *TaABCB1*

Wheat aneuploid stock consisting of 21 NT, 14 DT, and group 7 deletion lines was used for inter- and intra-chromosomal mapping of *TaABCB1*. A 150 double haploid Steptoe × Morex population^59^ was used for genetic mapping in barley (*Hordeum vulgare* L.). Plant genomic DNA was extracted following protocol by Sandhu^6^°. Gel blot analysis was performed using 10 µg of genomic DNA digested with *Hin*dIII and was size separated on 0.8% agarose gels. All steps including probe preparation, hybridization, and autoradiography for the gel blot DNA analysis were described previously^61^. The JoinMap® 4 was used to prepare the genetic linkage map^62^. The sequence of *HvABCB1* for physical localization on barley chromosome was taken from Dhaliwal *et al*., 2014.

### Cloning full-length genomic and cDNA copies of *TaABCB1*

The full-length *TaABCB1* sequence^38^ was used as a query to retrieve the ancestral sequences of *Triticum urartu* (TGAC_WGS_urartu_v1_contig_232156), *Aegilops speltoides* (TGAC_WGS_speltoides_v1_contig_201698) and *Aegilops tauschii (TGAC_WGS_tauschii_v1_contig_143178)* using BLASTn with default parameters. Homoeolog-specific primers were designed with terminal *att*B sites from the above three sequences and their specificity was tested on the NT lines. TaABCB1-4F and TaABCB1-4R (Supplementary Table S1) were used to amplify copies from *B* and *D* genomes and TaABCB1-4F and TaABCB1-6R to amplify the *A* copy. The homoeologs cDNA copies were cloned from CS stem mRNA. Primers TaABCB1-4F and TaABCB1-6R amplified the *A* copy. For each of the *B* and *D* copies, two pairs of primers (TaABCB1-4F and TaABCB1-5R; TaABCB1-5F and TaABCB1-4R) were designed to amplify two overlapping regions to clone full-length cDNA copies. Similarly, the promoter was amplified using forward and reverse primers of TaABCB1-Apromoter for *A*, TaABCB1-Bpromoter for *B*, and TaABCB1-Dpromoter for *D* copies. The 3′ untranslated Regions (3′ UTR) of homoeologs were amplified by using the common primers -TaABCB1-3UTR-F and -R. The genomic, promoter and cDNA fragments were amplified using PrimeSTAR GXL DNA Polymerase (Catalog #R050A, Clontech laboratory, CA, USA) in a PCR reaction (25 µl) composed of 100 ng genomic DNA or 50 ng of cDNA following manufacture recommendations. The PCR fragments were purified with 30% PEG 8000/30 mM MgCl_2_ and were cloned in pDONR^®^201 vector using BP Clonase II Enzyme Mix (Catalog#11789–020, Life Technologies, NY, USA) as per the manufacturer’s instructions. Clones with desired insert size were identified by *Bsr*GI restriction digestion. Three clones for each of the cloned fragments were sequenced using Eurofins MWG Operon Simple-Seq services, and data was analyzed by using ClustalW2 (http://www.ebi.ac.uk/Tools/msa/clustalw2/). The exon/intron junctions were predicted by aligning sequences of genomic copies with their respective cDNA.

### *In-silico* protein, promoter and ligand docking analysis

The predicted protein sequence were analyzed using ProtParam (http://web.expasy.org/protparam/) to compute various physical and chemical parameters including the molecular weight, theoretical pI, amino acid (aa) composition, instability index, aliphatic index and grand average of hydropathicity (GRAVY). The sequences (≈1000 bp upstream of ATG) cloned by homoeologous copy specific primers (as mentioned above) were scanned for auxin responsive elements (of the known motif) and other *cis*-elements using PlantCARE^63^ database with default settings. The number of copies of a motif was accounted by the occurrence of that motif in a particular promoter sequence. Docking of auxin (IAA molecule) on the predicted 3-D structure of TaABCB1 was generated using AutoDock vina in PyRx virtual screening software^64^ using default parameter. The docking results were visualized using the PyMOL.

### RNA extraction and cDNA preparation

The total RNA was isolated from leaf, root, crown, nodes, internodes, and flowering buds of the main tiller from cv. Indian at 2^nd^, 4^th^, 5^th^, 6^th^, 7^th^, and 8^th^ weeks (Feekes scale 2–9) using the hot phenol RNA extraction following Bennypaul *et al*., 2012. For 2^nd^ and 4^th^ week, RNA was extracted from the leaf whorl as no proper stem was formed. First strand cDNA was synthesized using Transcriptor First Strand cDNA Synthesis Kit using 1 μg of total RNA as per manufacturer’s recommendations (Cat #04896866001, Roche Diagnostics, USA).

### Single-strand conformation polymorphism (SSCP)

The SSCP analysis for *TaABCB1* homoeolog-specific expression pattern was performed as described by^66^. SSCP was performed to distinguish and differentiate the expression of homoelogous in different tissues based on the electrophoretic mobility of single-stranded DNA even in the presence of single base change. The product was amplified by TaABCB1-2F and -2R from cDNA of different tissues and CS-NT lines using Advantage® PCR Kits Polymerase mixes (Clontech, Catalog #639101). The PCR product was mixed with an equal volume of sequencing gel loading buffer (95% formamide, 20 mM EDTA, 10 mM NaOH, 0.05% bromophenol blue, and 0.05% xylene cyanol) and was denatured at 94 °C for 5 minutes. About 5 μl of the mixture was loaded onto 0.4 mm thick denaturing 8% polyacrylamide/8 M urea gel^67^. The gel was transferred to a blotting paper, dried in Bio-Rad gel drier and exposed to X-ray film for three to seven days and bands were scored manually.

### Quantitative real-time expression analysis

Quantitative reverse transcriptase real-time PCR (qRT-PCR) to analyze transcript levels in different tissues were performed using the LightCycler® 480 SYBR Green-I Master mix (Cat #04707516001) on Roche *LightCycler® 480* (Roche Diagnostics, USA). For homoeolog specific expression, forward and reverse primers TaABCB1-29 were used for the *A*, TaABCB1-30 for *B*, and TaABCB1-27 for *D* copies (Supplementary Table S1). The genome specificity (on NT lines and by sequencing) and melting curve analysis (Supplementary Fig. S12) was performed for each primer pair to ensure the detection of the desired product. PCR reaction consisted of 50 ng of cDNA, 100 nM of each primers, 3 mM of MgCl_2_, and 1X of SYBR Green-I Master mix in a total volume of 10μl. PCR conditions were 95 °C for 4 min, 4 cycles (95 °C for 1 min, 62 °C for 1 min and 72 °C for 1 min), 35 cycles (95 °C for 1 min, 58 °C for 1 min, 72 °C for 1 min) and 72 °C for 10 minutes. The values from three replicates of each sample were used for calculations and presented as Actin normalized individual data points using the ΔC_T_ method where ΔC_T_ = (C_T_ gene of interest − C_T_ internal control)^68^.

### *In*-*silico* Expression analysis

The *in*-*silico* expression for the *TaABCB1* homoeologs was calculated using transcriptome data from wheat expression database (http://wheat.pw.usda.gov/WheatExp/)^69^ using the amino acid sequence as a query against the TGACv1 assembly. The homoeologous copy expression among different tissues under normal conditions was plotted as TPM (Transcripts Per Kilobase Million) values.

### Virus-induced gene silencing

The VIGS was performed using oligos TaABCB1-200 and TaABCB1-224 as previously described^65^. In brief, two oligos with *Not*I and *Pac*I sites (Supplementary Fig. S11) were cloned into pBSMVγ vector using T4 DNA ligase. Additionally, a hairpin construct, TaABCB1-oligo consisting of an inverted repeat of 41 bp designed from wheat EST CJ800530 was also cloned. For each VIGS reaction, the three plasmids (pBSMVα, pBSMVβΔβa and pBSMVγ) were linearized and mixed in equimolar ratio to get about 1 μg of the mixture. Infectious RNAs were transcribed using the mMessage mMachine T7 *in-vitro* transcription kit (Cat# AM1344, Ambion, Austin, TX, USA), as per manufacturer’s recommendations. To target *TaABCB1* during stem elongation, two-week-old wheat plants were inoculated by rubbing the infectious RNA mixed with 45 μl of FES buffer on the second leaf using gentle strokes. The pγMCS was used as a ‘virus only’ control, pγPDS (*Phytoene desaturase*) as a positive control, and FES buffer (abrasive agent for rubbing) as a negative control. Viral and PDS symptoms were recorded for successful VIGS inoculation. Data on plant height of the main tiller from the soil surface to the tip of a spike was recorded at maturity.

### Stable RNAi transformation

The oligo TaABCB1-424 flanked by *att*B sites was ligated into pDONR®201 vector using the Gateway BP cloning system. The clones carrying the insert were identified by size separation on gel and clone identity was confirmed by sequencing. The confirmed inserts were transferred to hairpin RNAi Destination Clone pHellsgate 8 vector using the LR reaction. Clones carrying the expected insert size were identified by restriction-digestion and orientation of the construct was confirmed by DNA sequencing. Sequence verified vector was cloned in *Agrobacterium* strain C58C1 by electroporation and transferred to cultivar Bobwhite, following the protocol by^70^. Regenerated plants were transferred to a growth chamber and DNA from the 108 putative transformants was isolated using the CTAB method^60^. Integration of the transformants were confirmed using four different primer combinations (Supplemental Table 1) targeting different parts of the vector sequence. DNA amplifications were performed in a 10μl reaction containing 50 ng genomic DNA, 200 mM dNTPs, 1pmol primers in a PCR conditions of 94 °C for 5 min, followed by 4 cycles of 94 °C for 30 sec, 62 °C for 45 sec, 72 °C for 1 min, followed by 35 cycles of 94 °C for 30 sec, 58 °C for 45 sec, 72 °C for 1 min and 72 °C for 10 minutes. PCR product was separated on 1% agarose gel and expected band was scored relative to Bobwhite + vector control (10^6^:1).

### Phenotypic analysis of RNAi plants

The plant height of six TaABCB1T2-107 transformed plants was recorded from the soil surface to the tip of the spike. Individual internodes of a plant were recorded from the top of one node to the base of next node with peduncle being counted as internode 1. For coleoptile and root length measurements, a modified germination paper test^71^ was used. In brief, twelve seeds for each of the four independent TaABCB1T2 plants were placed in the middle of the moist germination paper (Heavy Germination paper #SD 7615 L), about one centimeter apart with germ end down. Germination paper was rolled in the form of a cigar and placed upright in a beaker at 4°C for 24hrs in dark to break dormancy and then to room temperature (23°C). Data on coleoptile length and root length were recorded on 7^th^ day to the nearest millimeter. The 12 seedlings from each of the four independent TaABCB1T2-107 plants was used to calculate the mean coleoptile and root length. Spikelet number, spike length, flag leaf length, and flag leaf width of the main tiller of the seven plants of TaABCB1T2-107 plants were recorded on matured plants. Flag leaf width was recorded at the maximum expansion near to the middle of the leaf. The seed weight of 100 seeds from each of five TaABCB1T2-107 plants and Bobwhite were record individually. Average of five plants was used to calculate the mean seed weight in grams except for 100 seed weight of one plant for C-Ph1 as transformation control. Mean of 20 seeds each from five TaABCB1T2-107 plants was photographed and processed with ImageJ^72^ to measure the seed length and breadth to the nearest millimeter. Data was analyzed and compared by one-way ANOVA at significant level of p < 0.05. When significant differences were detected, Tukey’s post-hoc test was used.

## Supplementary information

Supplementary Info
