# Supplementary material for: Molecular Characterization of Auxin Efflux Carrier- ABCB1 in hexaploid wheat
Source: Sci Rep. 2019 Nov 22;9:17327. doi: 10.1038/s41598-019-51482-5 (PMC6874703; doi:10.1038/s41598-019-51482-5)
Supplement: Supplementary file 1 — Supplementary Info [file 41598_2019_51482_MOESM1_ESM.pdf]

Molecular Characterization of Auxin Efflux Carrier- *ABCB1* in hexaploid wheat

Amita Mohan¶, Amandeep K Dhaliwal¶, Ragupathi Nagarajan, and Kulvinder S Gill*
Department of Crop and Soil Sciences, Washington State University, Pullman, WA-99164
¶These authors contributed equally to this work.

Amita Mohan: amitamohan@wsu.edu
Amandeep Dhaliwal: adhaliwal@wsu.edu
Ragupathi Nagarajan: ragupathi.nagarajan@wsu.edu
*Kulvinder S Gill: ksgill@wsu.edu; +1-509-335-4666

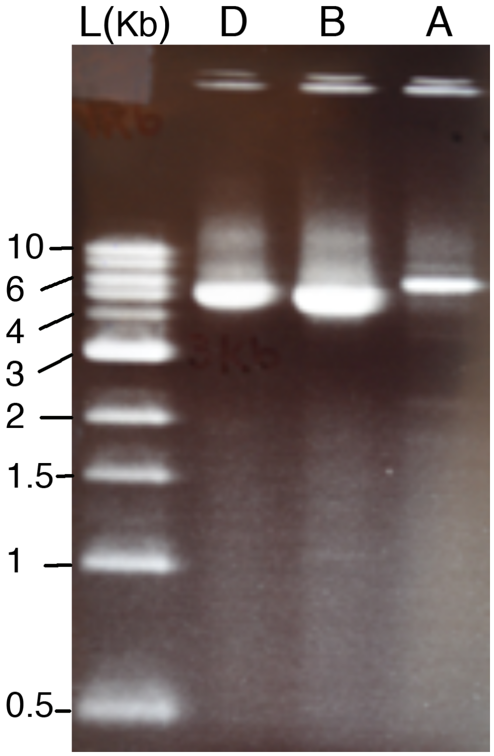

Supplementary Fig. S1: Cloning of homoeologous copies of *TaABCB1*. L represent molecular weight standard in Kb (10Kb NEB Ladder), A, B and D represent the homoeologous genomic copies of *TaABCB1*.

```
TaABCB1-A     ATGTCTAGCGACCCTGAAGAGATCAAGGCTCGCGTCGTCGTCCACGGCG-----------     49
TaABCB1-B     ATGTCTAGCGACCCTGAAGAGATCAAGGCTCGCGTCGTCGTCCACGGCGCCGACGCCGAC     60
TaABCB1-D     ATGTCTAGCGACCCTGAAGAGATCAAGGCTCGCGTCGTCGTCCACGGCGCCGGCGCCGAC     60
              *************************************************

TaABCB1-A     -CCGACGCCGCCGACGAGTGGGCCCGCCCGGAGCTCGAGGCCTTCCACCTCCCCTCCACC     108
TaABCB1-B     GCCGACGCCGACGCCGAGTGGGCCCGCCCGGAGCTCGAGGCCTTCCACCTCCCCTCCACC     120
TaABCB1-D     GCCAACGCCGCCGACGAGTGGGCCCGCCCGGAGCTCGAGGCCTTCCACCTCCCCTCCACC     120
               ** ****** ** **********************************************

TaABCB1-A     TCCCAGCCCCCACACTTGTTCCACCCACCGCACCCAGAGCCAGAAGCAGCAGAGCAATCC     168
TaABCB1-B     TCCCAGCCCCCACACTTGTTTCACCCACCGCACCCGGAGCCAGAAGCAGCAGAGCAATCC     180
TaABCB1-D     TCCCAGCCCCCGCACTTGTTCCACCCACCGCACCCAGAGCCAGAAGCAGCAGAGCAATCC     180
              *********** ******** ************** ************************

TaABCB1-A     ACGCCAGCACCGGCCGTGGCAGCAGCTACCACCA---ACAATGCGTCTGGTTCCCCCCCT     224
TaABCB1-B     ACGCCAGCACCGGCCGTCGCAGCAGCTACC---ACCAACAATGCGTCAGGTTCCCCCCCT     237
TaABCB1-D     ACGCCAGCACCGGCCGTGGCAGCAGCTACCGGCACCAACAATGCGTCTGGTTCCCCCCCT     240
              ***************** ************   *   ********** ************

TaABCB1-A     ------------CCTCCCCCCGCGCCACTGGAGACGGAGCAGCTTCCGCCCAATGCCAAG     273
TaABCB1-B     CCTCCTCGCTCGCCGCCTCCCGCGCCACTGGAGACGGAGCAGCTTCCGCCCAATGCCAAG     297
TaABCB1-D     CCTCCTCGCTCGCCGCCTCCCGCGCCACTGGAGACGGAGCAGCTTCCGCCCAATGCCAAG     300
                          ** ** ******************************************

TaABCB1-A     CCGCCTGCCGACGAGAAGCCGCCCCCTCCGGCGCCGGCGGCCGCCCTGCGCGACTTGTTC     333
TaABCB1-B     CCGCCTGCCGACGAGAAGCCGCCCCCTCCGGCGCCGGCGGCCGCTCTGCGCGACTTGTTC     357
TaABCB1-D     CCGCCTGCCGACGAGAAGCCGCCCCCTCCGGCGCCGGCGGCCGCCCTGCGCGACTTGTTC     360
              ******************************************** ***************

TaABCB1-A     CGCTTCGCCGACGGCCTAGATCGCGTCCTCATGGCCGTGGGCACGCTCGGCGCCCTCGTC     393
TaABCB1-B     CGCTTCGCCGACGGCCTAGATCGCGTCCTCATGGCCGTGGGCACGCTCGGCGCTCTCGTC     417
TaABCB1-D     CGCTTCGCCGACGGCCTAGATCGCGTCCTCATGGCCGTGGGCACGCTCGGCGCCCTCGTC     420
              ***************************************************** ******

TaABCB1-A     CACGGCTGCTCCCTCCCCGTCTTCCTCCGCTTCTTCGCCGACCTCGTCGACTCCTTCGGC     453
TaABCB1-B     CATGGCTGCTCCCTCCCCGTCTTCCTCCGCTTCTTCGCCGACCTCGTCGACTCCTTCGGC     477
TaABCB1-D     CACGGCTGCTCCCTCCCCGTCTTCCTCCGCTTCTTCGCCGACCTCGTCGACTCCTTCGGC     480
              ** *********************************************************

TaABCB1-A     TCCCACGCCGACGACCCGGACACCATGGTCCGCCTCGTCGTGAAGTACGCCTTCTACTTC     513
TaABCB1-B     TCCCACGCCGACGACCCGGACACCATGGTCCGCCTCGTCGTCAGGTACGCCTTCTACTTC     537
TaABCB1-D     TCCCACGCCGACGACCCGGACACCATGGTCCGCCTCGTCGTCAAGTACGCCTTCTACTTC     540
              ***************************************** * ****************

TaABCB1-A     CTCGTCGTGGGCGCCGCCATCTGGGCGTCGTCCTGGGCCGAGATTTCCTGCTGGATGTGG     573
TaABCB1-B     CTCGTCGTCGGCGCCGCCATCTGGGCGTCGTCCTGGGCCGAGATTTCCTGCTGGATGTGG     597
TaABCB1-D     CTCGTCGTGGGCGCCGCCATCTGGGCGTCGTCCTGGGCCGAGATTTCCTGCTGGATGTGG     600
              ******** ***************************************************

TaABCB1-A     ACCGGCGAGCGGCAGTCCACTCGGATGCGGATCCGGTACCTCCAGGCGGCGCTCAAGCAG     633
TaABCB1-B     ACCGGCGAGCGGCAGTCCACTCGGATGCGGATCCGGTACCTCCAGGCGGCGCTCAAGCAG     657
TaABCB1-D     ACCGGCGAGCGGCAGTCCACCCGGATGCGGATCCGGTACCTCCAGGCGGCGCTCAAGCAG     660
              ******************** ***************************************

TaABCB1-A     GACGTCTCCTTCTTCGACACCGACGTGCGCACCTCCGACGTCATCTATGCCATCAACGCC     693
TaABCB1-B     GACGTCTCCTTCTTCGACACCGACGTGCGCACCTCCGACGTCATTTATGCCATCAACGCC     717
TaABCB1-D     GACGTCTCCTTCTTCGACACCGACGTGCGCACCTCCGACGTCATCTATGCCATCAACGCC     720
              ******************************************** ***************

TaABCB1-A     GACGCTGTCATGGTCCAGGACGCCATCAGCGAGAAGCTCGGCAACCTCATCCACTACATG     753
TaABCB1-B     GACGCTGTCATCGTCCAGGACGCCATCAGCGAGAAGCTCGGCAACCTCATCCACTACATG     777
TaABCB1-D     GACGCTGTCATCGTCCAGGACGCCATCAGCGAGAAGCTCGGCAACCTCATCCACTACATG     780
              *********** ************************************************

TaABCB1-A     GCCACCTTCGTCGCCGGCTTCGTCGTCGGCTTCACCGCAGCGTGGCAGCTCGCGCTCGTC     813
TaABCB1-B     GCCACCTTCGTCGCCGGCTTCGTCGTCGGCTTCACCGCAGCGTGGCAGCTCGCGCTCGTC     837
TaABCB1-D     GCCACCTTCGTGGCCGGCTTCGTCGTCGGCTTCACCGCAGCGTGGCAGCTCGCGCTCGTC     840
              *********** ************************************************

TaABCB1-A     ACGCTCGCCGTCGTGCCGCTCATCGCCGTCATCGGCGGCCTCACCGCCGCCACCATGGGC     873
TaABCB1-B     ACGCTCGCCGTCGTGCCGCTCATCGCCGTCATCGGCGGCCTCACCGCCGCCACCATGGGC     897
TaABCB1-D     ACGCTGGCCGTCGTGCCGCTCATCGCCGTCATCGGCGGCCTCACCGCCGCCACCATGGGC     900
              ***** ******************************************************

TaABCB1-A     AAGCTCTCCTCCAAGAGCCAGGACGCGCTGTCCAGCGCCAGCAACATCGCGGAGCAGGCC     933
TaABCB1-B     AAGCTCTCCTCCAAGAGCCAGGACGCGCTGTCCAGCGCCAGCAACATCGCGGAGCAGGCC     957
TaABCB1-D     AAGCTCTCCTCCAAGAGCCAGGACGCGCTGTCCAGCGCCAGCAACATCGCGGAGCAGGCC     960
              ************************************************************
```

```
TaABCB1-A      CTGTCGCAGATACGAGTCGTGCAGTCGTTCGTGGGTGAGGAGCGGGTGGCGCAGGCCTAC      993
TaABCB1-B      CTGTCGCAGATACGGATCGTGCAGTCATTCGTGGGTGAGCAGCGGGTGGCGCAGGCCTAC      1017
TaABCB1-D      CTGTCGCAGATACGGATCGTGCAGTCGTTCGTGGGTGAGCAGCGGGTGGCGCAGGCCTAC      1020
               **************  ********** ************ ********************

TaABCB1-A      TCGGCGGCGCTAGCCGTGGCGCAGAGCATCGGCTACCGGAACGGCTTTGCCAAGGGCCTC      1053
TaABCB1-B      TCGGCGGCGCTAGCCGTGGCGCAGAGCATCGGCTACCGGAACGGCTTTGCCAAGGGCCTC      1077
TaABCB1-D      TCGGAGGCGCTAGCCGTGGCGCAGAGCATCGGCTACCGGAACGGCTTTGCCAAGGGCCTC      1080
               **** *******************************************************

TaABCB1-A      GGGCTGGGCGGCACCTACTTCACCGTCTTCTGCTGCTACGCTCTGCTCCTCTGGTACGGC      1113
TaABCB1-B      GGGCTGGGCGGCACCTACTTCACCGTCTTCTGCTGCTACGCTCTGCTCCTCTGGTACGGC      1137
TaABCB1-D      GGGCTGGGCGGCACCTACTTCACCGTCTTCTGCTGCTACGCTCTGCTCCTCTGGTACGGC      1140
               ************************************************************

TaABCB1-A      GGGCACCTCGTTCGCGGCCACCACACCAACGGCGGGCTGGCCATCGCCACCATGTTCTCC      1173
TaABCB1-B      GGGCACCTCGTTCGCGGCCACCACACCAACGGCGGGCTGGCCATCGCCACCATGTTTTCC      1197
TaABCB1-D      GGGCACCTCGTTCGCGGCCACCACACCAACGGCGGGCTGGCCATCGCCACCATGTTCTCC      1200
               ******************************************************** ***

TaABCB1-A      GTCATGATCGGCGGACTGTAAGTCTTCCGTCTTATTACTCCTCTCCTCTCCTCTTTATTT      1233
TaABCB1-B      GTCATGATCGGCGGACTGTAAGTCGTCTTATTACT----CCTCTCCTCTT----------      1243
TaABCB1-D      GTCATGATCGGCGGACTGTAAGTCTTCCGTCTTATTACTCCTCTCCTCTCCTCTCCTCCT      1260
               ************************ **    *  *    **********

TaABCB1-A      TTTTATTTATAAGGAGAGGAGAGGACACCTTGCCGCCGCTGAAAGGGAAGATGCATTGAT      1293
TaABCB1-B      TATTTAT--TGAAGGACTGAAAGATGGCATTTCTAGTCAGTTGTGACATTTTGTATTGAT      1301
TaABCB1-D      TATTTAT--TTA--TAAGGAGAGGACACCTTGGCGCCGCTGAAAGGGAAGATGCATTGAT      1316
               * **  *  * *      ** **    * **             *  *   ** ******

TaABCB1-A      GGATTCTTGTGTTGTGCGAGCAGCGGTCTTGGGCAGTCGGCGCCGAGCATGGCGGCGTTT      1353
TaABCB1-B      GGATTGTTGTGTTTGCGCGACAGCGCTCTTGGGCAGTCGGCGCCGAGCATGGCGGCGTTT      1361
TaABCB1-D      GGATTCTTGTGCTGTGCGAGCAGCGGTCTTGGGCAGTCGGCGCCGAGCATGGCGGCGTTT      1376
               ***** ***** *       ***** **********************************

TaABCB1-A      GCCAAGGCGAGGGTTGCGGCCGCGAAGATCTTCCGTATCATCGACCACACGCCAGGCATC      1413
TaABCB1-B      GCCAAGGCGAGGGTTGCGGCCGCGAAGATCTTCCGTATCATCGACCACACGCCAGGCGTC      1421
TaABCB1-D      GCCAAGGCGAGGGTTGCGGCCGCGAAGATCTTCCGTATCATCGACCACACGCCAGGCATC      1436
               ********************************************************* **

TaABCB1-A      ACCAAAGAAGGCGTAGAGCTGGAGTCGGTGACCGGGCGTCTGGAGCTGAGGAACGTGGAG      1473
TaABCB1-B      ACCAAGGAAGGCGTGGAGCTGGAGTCGGTGACCGGGCGGCTGGAGCTGAGGAACGTGGAG      1481
TaABCB1-D      ACCAAGGAAGGCGTGGAGCTGGAGTCGGTGACGGGGCGGCTGGAGCTGAGGAACGTGGAG      1496
               ***** ******** ***************** ***** *********************

TaABCB1-A      TTCGCGTACCCGTCCCGGCCGGACACGCCGATTCTGCGCCGCTTCTCTCTAAGCGTACCG      1533
TaABCB1-B      TTCGCGTACCCGTCCCGGCCGGACACGCCGATTCTGCGCCGCTTCTCTCTAAGCGTACCG      1541
TaABCB1-D      TTCGCGTACCCGTCCCGGCCGGACACGCCGATTCTGCGCCGCTTCTCTCTAAGCGTACCG      1556
               ************************************************************

TaABCB1-A      GCCGGGAAGACGATCGCTCTGGTTGGCAGCTCTGGCTCCGGGAAGAGTACGGTGGTGTCC      1593
TaABCB1-B      GCAGGGAAGACGATCGCTCTGGTTGGCAGCTCTGGCTCCGGGAAGAGTACGGTGGTGTCC      1601
TaABCB1-D      GCCGGGAAGACGATCGCTCTGGTTGGCAGCTCTGGCTCCGGGAAGAGTACGGTGGTGTCG      1616
               ** ********************************************************

TaABCB1-A      CTGATCGAGAGGTTCTACGACCCGAGTTCAGGTCTGACTGACTCTCAAGTTTTCTTTCTT      1653
TaABCB1-B      CTGATCGAGAGGTTCTACGACCCGAGTTCAGGTCCGACTCTCAAGTTTTCTTTCTT-ACT      1660
TaABCB1-D      CTGATCGAGAGGTTCTACGACCCGAGTTCAGGTCTGACTGACTCTCAAGTTTTCCCTTGT      1676
               ********************************** ****  *       ****     *

TaABCB1-A      ACTCGATTATTAATTTACATTACATTTGGAAGGAGTGAGTTAGTAGGCGCTTGTTAAAAT      1713
TaABCB1-B      CG----ATTATAATTTACATTACATTTGGAAGGAGTGAGTTAGTAGGCGCTTGTCAAAAT      1716
TaABCB1-D      CT----CTTACTCGAGTATAATTTACTGGAAGGAGTGAGTTAGTAGGCGCTTGTTAAAAT      1732
                      *                  **************************** *****

TaABCB1-A      GCGAGCAGGGCAAATCATGCTTGACGGTGTCGAGCTCAAGGATCTGAAGCTGCGGTGGCT      1773
TaABCB1-B      GCGAGCAGGGCAAATCATGCTTGACGGTGTTGAGCTCAAGGATCTGAAGCTGCGGTGGCT      1776
TaABCB1-D      GCGAGCAGGGCAAATCATGCTTGACGGTGTTGAGCTCAAGGATCTGAAGCTGCGGTGGCT      1792
               ****************************** *****************************

TaABCB1-A      GCGGTCGCAGATCGGCCTGGTGAGCCAGGAGCCGGCGCTGTTCGCGACGAGCATCAGGGA      1833
TaABCB1-B      GCGGTCGCAGATCGGCCTGGTGAGCCAGGAGCCGGCGCTGTTCGCGACGAGCATCAGGGA      1836
TaABCB1-D      GCGGTCGCAGATCGGCCTGGTGAGCCAGGAGCCGGCGCTGTTCGCGACGAGCATCAGGGA      1852
               ************************************************************

TaABCB1-A      GAACCTGCTGCTTGGGAGGGAGGAGGCGAGCCAGGTGGAGATGGAGGAGGCCGCCAGGGT      1893
TaABCB1-B      GAACCTGCTGCTTGGGAGGGAGGAGGCGAGCCAGGTGGAGATGGAGGAGGCCGCCAGGGT      1896
TaABCB1-D      GAACCTGCTGCTTGGGAGGGAGGAGGCGAGCCAGGTGGAGATGGAGGAGGCCGCCAGGGT      1912
               ************************************************************
```

```
TaABCB1-A     CGCCAACGCCCACTCCTTCATCATCAAGCTCCCCGACGGCTACGACACGCAGGTCCGTCC     1953
TaABCB1-B     CGCCAACGCCCACTCCTTCATCATCAAGCTCCCCGACGGCTACGACACGCAGGTCCGTCC     1956
TaABCB1-D     CGCCAACGCCCACTCCTTCATCATCAAGCTCCCCGACGGCTACGACACGCAGGTCCGTCC     1972
              ************************************************************

TaABCB1-A     GTCCGTTGGTCACTCCGGCAATCATATCATATTAGATTCATCCCGCGCCAGCGCCGCGCG     2013
TaABCB1-B     GTTGGTCACTCCGGCAATCATA------------TCATCACCCC--GCGCCGCGCGTGCT     2002
TaABCB1-D     GTCCGTCCGTTGGTCACTCCGG------------CAATCATATTAGATTCATCCCGCGCG     2020
              **  **   *    *   *                  ***              ** **

TaABCB1-A     TGCTAGCTTCTTCCTTTTTATCCGCCCGATCCGTGCGCCATTAGGCT---------GGCA     2064
TaABCB1-B     AGCTAGCTTCTTCCTTTTTATCCGCCCGATCCATGCGCCATTAGGCTTGGCATAGTAGAT     2062
TaABCB1-D     TGCTAGCTTCTTCCTTTTTATCCGCCCGATCCGTGCGCCATTAGGCT---------GGAG     2071
               ******************************* **************          *

TaABCB1-A     TAGTAGATAGCTCTGCCCTTTCCACCATCCTTTTTTCCTCATCACACCCATCA-----TT     2119
TaABCB1-B     AGATAGATAGCTTTGCCCTTTCCACCATCCTTTTTTCCTCATCACACCCATCCCATCATT     2122
TaABCB1-D     TAGTAGATAGCTTTGCCCTTTCCACCATCCTTTTTCCCTCATCACACCCATCCCATCATT     2131
                 ********* ********************** ****************      **

TaABCB1-A     GGCCCCTTTG---CCGTGGAGTAGATAGAGGAGAGTAGCTAGCTAGATACCCCCGGCTCA     2176
TaABCB1-B     GGCCCCTTTGCCGTGGAGGAGTTGATAGAGGAGAGTAGCTAGCTAGATACCCCCGGCTCA     2182
TaABCB1-D     GGCCCCTTTGCCGTGGAGGAGTAGATAGAG---------GAGCTAGATACCCCCGGCTCA     2182
              **********     * ***** *******          ********************

TaABCB1-A     GATCGGATGCTTGGTGCTCCATTTGTTTGTTTGTTAGGCAACAACATCACATGATGACAC     2236
TaABCB1-B     GATCGGATGCTTGGTGCTCCATTTGTTTGTTTGTTAGGCAACAACATCACATGATGACAC     2242
TaABCB1-D     GATCGGATGCTTGGTGCTCCATTTGTTTGTTTGTTAGGCAACAACATCACATGATGACAC     2242
              ************************************************************

TaABCB1-A     CTTCTCTACTGGGCTCCATCACAGTCACAGGGCCCATTGTTACTACTACCTCCGTGTAGG     2296
TaABCB1-B     CTTCTCTACTGGGCTCCATCACAGGGCCCAT-----TTGTTACTAGTGCAATACTATTTC     2297
TaABCB1-D     CTTCTCTACTGGGCTCCATCACAGTCACAGGGCCCATTGTTACTAGTGCAATACTATTTC     2302
              ************************   *        ********* * *     * *

TaABCB1-A     TGAATAAATCACTCGCGTGGTTCTAGATCATCGATTTGAGGAATTAAATATGTGTTATAT     2356
TaABCB1-B     ACCACACCACGAAGAAGAA-----------------------------------------     2316
TaABCB1-D     AC-----CACCAAGAAGAA-----------------------------------------     2316
                       *      *

TaABCB1-A     GTCATGAAAAGTATATCACTAGATTTTTACAATGATGTAGTTTTTAAATACATATCTTTT     2416
TaABCB1-B     ------------------------------------------------------------     2316
TaABCB1-D     ------------------------------------------------------------     2316

TaABCB1-A     GTCACATATAATACATATTTAGATAGTTAAATCGTCAACCTAGAACTACACGAATGACTT     2476
TaABCB1-B     ------------------------------------------------------------     2316
TaABCB1-D     ------------------------------------------------------------     2316

TaABCB1-A     ATTCACCGAGACAGAGGTAGTAATGCAATACTATTTCACCACGAAGAAGAAGAAGCAATT     2536
TaABCB1-B     ------------------------------------------GAAGAAAAAGAAGCTATT     2334
TaABCB1-D     ------------------------------------------GAAGAAGAAGAAGCAATT     2334
                                                        ****** ******* ***

TaABCB1-A     GTTTGTTTGTTAGAGCAAGTACAATAGAGCTGAGTTAGCGGACTATAAGGAATAAACTAG     2596
TaABCB1-B     GTTTGTTTGTTTGTTAGGAGCATGTTGCTTTAAA--------------------------     2368
TaABCB1-D     GTTTGTTTGTTA----GGAGCATGTTGCTTTACATTGC----------AAAAAAAAGGAG     2380
              ***********             * *   *

TaABCB1-A     TATATTTTTGCTTAGTTGGAGGAAGGAGAAGATGAGAGAGAAGGTAAGCGGGCTCTTTGT     2656
TaABCB1-B     ------------------------------------------------------------     2368
TaABCB1-D     CA---TGTTG--------------------------------------------------     2387

TaABCB1-A     GAAGAGCGGGCTCTAGCACGTGTTCCTAGACGCTTTGTGAGAATGAAATGTGGGCCACAT     2716
TaABCB1-B     ------------------------------------------------------------     2368
TaABCB1-D     ------------------------------------------------------------     2387

TaABCB1-A     AATAAAAAAATAGTACACTCTTTTGATCTACTATTGTACATGTTGACTATAAGATGGCTT     2776
TaABCB1-B     ------------------------------------------------------------     2368
TaABCB1-D     ------------------------------------------------------------     2387

TaABCB1-A     GTAGATGACATGGCACTGGCTTATAGCCAGCAGCTGACTATACTATTAACCATGCTCTTA     2836
TaABCB1-B     ------------------------------------------------------------     2368
TaABCB1-D     ------------------------------------------------------------     2387
```

```
TaABCB1-A      TAGGAGCATGTTGCTTTAAATAAATGCTGTTAGGAGTTGGTAACAGTTAGTA----CGTA      2892
TaABCB1-B      ---------------T--AAATGCTGCTGTTAGGAGTTGGTA-------ACAGTTACGTA      2404
TaABCB1-D      --------ATTTAAATAAAATAAATGCTATTAGGAGTTGGTAACAGTTAGTACGTACCTG      2439
                              *  **    **** *************         *    * *

TaABCB1-A      CCTGCCTGCCTGCCTCCTCCATTTTCGAAAAGTCCCTTTCTCACCACAA-----------      2941
TaABCB1-B      CCAGCCTGCCTGCCTCCTCCATTTTCGAAAAGTCCCTTTCTCACCACAAACTAACCAACA      2464
TaABCB1-D      CCTGCCTGCCTGCCTCCTCCATTTTCCAAAAGTCCCTTTCTCACCAC-------GAAACA      2492
               ** *********************** ********************

TaABCB1-A      --------------GCAGCTGAGCCGAGAATGGACCATGATTTCACACCACTCTTTTGCT      2987
TaABCB1-B      CAACACAAGCAGCTGAGCCGAGCCCAAGAATAGGCCATGATTTCACACTACTCTTTTGCT      2524
TaABCB1-D      CAACACAAGCAGCTGAGGGGGAGCCGAGAATGGGCCATGATTTCACACTACTCTTTTGCT      2552
                             *        ** ***** * ************** ***********

TaABCB1-A      GCCTCTTTATTCCGCTCCAGCCCAGCAGTAAGGCTGGTCATAGTGGGGAGTAACTTATAC      3047
TaABCB1-B      GCCTCTTTATTCCGCTCCAGCCCAGCG---------------------------------      2551
TaABCB1-D      GCCTCTTTATTCCGCTCCAGCCCAGCA---------------------------------      2579
               **************************

TaABCB1-A      CAGTGTTGTGCATATAACACTAGTCTAAGTTATTATCTTCATAGTGCAAAGTAACTAAAT      3107
TaABCB1-B      ------------------------------------------------------------      2551
TaABCB1-D      ------------------------------------------------------------      2579

TaABCB1-A      AGTAATGTCATAGATGATTTCATTTAGTAGCTCATAGACTCATTGTACCACAAGCACCTT      3167
TaABCB1-B      ------------------------------------------------------------      2551
TaABCB1-D      ------------------------------------------------------------      2579

TaABCB1-A      TTTTCTCATTAAATAATTACCACATAACGAAATTATCTTGAAATATGTTAAGTTACTATC      3227
TaABCB1-B      ------------------------------------------------------------      2551
TaABCB1-D      ------------------------------------------------------------      2579

TaABCB1-A      TAAGTTACTTTACTATGGCTAGCCTAAGAGGCCTCCAGCAGTAAACTAATTTCAGGTCGG      3287
TaABCB1-B      ----------------------------------------GTAAACTAATTCCAGGTCGG      2571
TaABCB1-D      ----------------------------------------GTAAACTAATTCCAGGTCGG      2599
                                                       *********** ********

TaABCB1-A      CGTTTTGTCAGGAATGTCGAGTTGATCTCACAAGATTCAAGCATGCTTTGTCCAGTAGTA      3347
TaABCB1-B      CGTTTTGTCAGGAATGTCAAGTTGATCTCACAAGATTCAAGCATGCTTTGTCCACTAGTA      2631
TaABCB1-D      CGTTTTGTCAGGAATGTCAAGTTGATCTCACAAGATTCAAGCATGCTTTGTCCAGTAGTA      2659
               ****************** *********************************** *****

TaABCB1-A      CGTTCCTT--------------------TTATTATTTTTTCAGC------AGTGGCAGTG      3381
TaABCB1-B      GGAGTACGTCCGATGGTGCTTTTAGTTTTTATTATTTTTTCAGCAGTGGCAGTGGCAGTG      2691
TaABCB1-D      CG--------------------TTCCTTTTATTATTTTTTCAGCAGTGGCAGTGGCAGTG      2699
                *                          ****************      **********

TaABCB1-A      GCACAGCACTTACTTGACCAAAAGCTGCCATTGATAATTGTTGACATACGATGTGCTGGC      3441
TaABCB1-B      GCACAGCACTTATTTGACCAAAAGCTGCCACTGATAAT----------------------      2729
TaABCB1-D      GTACAGCACTTGCTTGACCAAAAGCTGCCATTGATAAT----------------------      2737
               * *********  ***************** *******

TaABCB1-A      TAATCTCTTTCATCAGATTAGTCTTTTAATTGCATTGGCTAAAGCACGCATTTTTACCTG      3501
TaABCB1-B      ------------------------------------------------------------      2729
TaABCB1-D      ------------------------------------------------------------      2737

TaABCB1-A      GTATCGGAGCCAAAAGATCTTGAGTTCGAGACCCAGCCTAATCTTTTTCATCAGATCGGT      3561
TaABCB1-B      ------------------------------------------------------------      2729
TaABCB1-D      ------------------------------------------------------------      2737

TaABCB1-A      TTTTTGGTTGCATTAACTAGACCATGCACTTGTACAATAATTGATCGAAGCAAGCACATG      3621
TaABCB1-B      -----------------------------------------TGATCCAAGCAAGCACATG      2748
TaABCB1-D      -----------------------------------------TGATCCAAGCAAGCACATG      2756
                                                        ***** *************

TaABCB1-A      GGCTACTGATCCAAGCTCCCTTCCCTATCCATCGACA---TCGACACCGATCCCATCACA      3678
TaABCB1-B      GGCTACTGACCCAAGCTCCCTTCCCTAGTACTCCATCGACACCGACCCCATCACGTGGGG      2808
TaABCB1-D      GGCTACTGATCCAAGCTCCCTTCCCTAGTACTCCATCGACACCGACCCCATCACATGG--      2814
               ********* *****************    ** *      *    ** *** * *

TaABCB1-A      TGGATGGCACCATGCTCTGGCTCTACTCCCAGTCAAAATTCCACCATAAACAATTCAATT      3738
TaABCB1-B      AAGATAGCACCATGCCCTGGCTCTACTCCCAGTCAAAATTCCACCATAAACAATTCTGTC      2868
TaABCB1-D      ---ATGGCACCATGCCCTGGCTCTACTCCCAGTCAAAATTCCACCATAAACAATTCAATT      2871
                  ** ********* ****************************************  *
```

```
TaABCB1-A      CTGT-CACATTCTACAAAATTAAGCTGGCCTCCAGTCAAAATTCCCACATAAACAAGCGC      3797
TaABCB1-B      ACATTCTACTAGTACAAAATTAAGCTGGCCTCCAGTCAAAATTCCCACATAAACAAGCGC      2928
TaABCB1-D      CTGT-CACATTCTACAAAATTAAGCTGGCCTCCAGTCAAAATTCCCATATAAACGACTCT      2930
                  * *   *  *********************************** ****** *

TaABCB1-A      ACATGCATACATACGACACGACCCTAGAGTAGTACTGACAAGTCAATATGTTTGAACATG      3857
TaABCB1-B      ACA----------------------TGCATAC-TGACAAAAGTCAATATGTTTGAACATG      2965
TaABCB1-D      AGA----------------------GGAGTACTACTGACAAGTCAATATGTTTGAACATG      2968
               * *                       *  **      * *********************

TaABCB1-A      GTGATACAGGTGGGGGAGCGCGGCCTGCAGCTCTCCGGCGGCCAGAAGCAGCGGATCGCC      3917
TaABCB1-B      GTGATACAGGTGGGGGAGCGCGGCCTGCAGCTCTCCGGCGGCCAGAGGCAGCGGATCGCC      3025
TaABCB1-D      GTGATACAGGTGGGGGAGCGCGGCCTGCAGCTCTCCGGCGGCCAGAAGCAGCGGATCGCC      3028
               ********************************************** *************

TaABCB1-A      ATCGCCCGTGCGATGCTCAAGAACCCGGCCATCCTCCTCCTGGACGAGGCCACCAGCGCG      3977
TaABCB1-B      ATTGCCCGTGCGATGCTCAAGAACCCGGCCATCCTCCTCCTGGACGAGGCCACCAGCGCG      3085
TaABCB1-D      ATTGCCCGTGCGATGCTCAAGAACCCGGCCATCCTCCTCCTGGACGAGGCCACCAGCGCG      3088
               ** *********************************************************

TaABCB1-A      CTGGACTCCGAGTCCGAGAAGCTCGTGCAGGAGGCGCTGGACCGCTTCATGATCGGCCGC      4037
TaABCB1-B      CTGGACTCCGAGTCGGAGAAGCTCGTGCAGGAGGCGCTGGACCGCTTCATGATCGGCCGC      3145
TaABCB1-D      CTGGACTCCGAGTCGGAGAAGCTCGTGCAGGAGGCGCTGGACCGCTTCATGATCGGCCGC      3148
               ************** *********************************************

TaABCB1-A      ACCACCCTTGTCATCGCGCACAGGCTCTCAACCATCCGCAAGGCCGACCTCGTTGCCGTC      4097
TaABCB1-B      ACCACCCTCGTCATCGCGCACAGGCTCTCCACCATCCGCAAGGCCGACCTCGTCGCCGTC      3205
TaABCB1-D      ACCACCCTGGTCATCGCGCACAGGCTCTCCACCATCCGCAAGGCCGACCTCGTCGCCGTC      3208
               ******** ******************** *********************** ******

TaABCB1-A      CTGCAGGCTGGCGCCGTGTCCGAGATGGGCGCGCACGACGACCTCATGGCCAGAGGGGAC      4157
TaABCB1-B      CTGCAGGCTGGCGCCGTCTCCGAGATGGGCGCGCACGACGACCTCATGGCCAGAGGGGAC      3265
TaABCB1-D      CTGCAGGCTGGCGCCGTGTCCGAGATGGGCGCGCACGACGATCTCATGGCCAGAGGGGAC      3268
               ***************** *********************** ******************

TaABCB1-A      AGCGGCGCGTACGCCAAGCTCATCCGCATGCAGGAGCAGGCGCACGAGGCGGCCATCGTC      4217
TaABCB1-B      AGCGGCGCGTACGCCAAGCTCATCCGCATGCAGGAGCAGGCGCACGAGGCGGCCCTCGTC      3325
TaABCB1-D      AGCGGCGCGTACGCCAAGCTCATCCGCATGCAGGAGCAGGCGCACGAGGCGGCCCTCGTC      3328
               ****************************************************** *****

TaABCB1-A      AGCGCCAGGAGGAGCAGCGCAAGGCCCTCCAGCGCCCGCAACTCCGTCAGCTCACCCATC      4277
TaABCB1-B      AGCGCCAGGAGGAGCAGCGCAAGGCCCTCCAGCGCCCGCAACTCCGTCAGCTCACCCATC      3385
TaABCB1-D      AGCGCTAGGAGGAGCAGCGCAAGGCCCTCCAGCGCCCGCAACTCCGTCAGCTCACCCATC      3388
               ***** ******************************************************

TaABCB1-A      ATGATGCGCAACTCCTCCTACGGCCGCTCGCCCTACTCCCGCCGCCTCTCCGACTTCTCC      4337
TaABCB1-B      ATGATGCGCAACTCCTCCTACGGCCGCTCGCTCTACTCCCGCCGCCTCTCCGACTTCTCC      3445
TaABCB1-D      ATGATGCGCAACTCCTCCTACGGCCGCTCGCCCTACTCCCGCCGCCTCTCCGACTTCTCC      3448
               ******************************* ****************************

TaABCB1-A      ACCGCCGACTTCAGCCTGTCCGTCATACATGACCCGGCCGCCCACCGGATGGGCATGGGC      4397
TaABCB1-B      ACCGCCGACTTCAGCCTGTCCGTCATACATGACCCGGCCGCCCACCGGATGGGCA-----      3500
TaABCB1-D      ACCGCCGACTTCAGCCTGTCCGTCATACATGACCCAGCCGCCCACCGGATGGGCATGGGC      3508
               *********************************** *******************

TaABCB1-A      A------TGGGAATGGAGAAGCTCGCGTTCCGTGCGCAGGCCAGCTCCTTCTGGCGGCTG      4451
TaABCB1-B      -------TGGGAATGGAGAAGCTCGCGTTCCGTGCGCAGGCCAGCTCCTTCTGGCGGCTG      3553
TaABCB1-D      ATGGGAATGGGAATGGAGAAGCTGGCGTTCCGTGCGCAGGCCAGCTCCTTCTGGCGGCTG      3568
                      **************** ************************************

TaABCB1-A      GCCAAGATGAACTCGCCCGAGTGGGGCTACGCGCTCGCCGGCTCCGTGGGGTCCATGGTG      4511
TaABCB1-B      GCCAAGATGAACTCGCCGGAGTGGGGCTACGCGCTCGCCGGCTCCGTGGGGTCCATGGTG      3613
TaABCB1-D      GCCAAGATGAACTCGCCCGAGTGGGGCTACGCGCTCGCCGGCTCCGTGGGGTCCATGGTA      3628
               ***************** *****************************************

TaABCB1-A      TGCGGCTCCTTCAGCGCTATCTTCGCTTACATCCTCAGCGCGGTGCTCAGCATCTACTAC      4571
TaABCB1-B      TGCGGCTCCTTCAGCGCCATCTTCGCCTACATCCTCAGCGCGGTGCTCAGCATCTACTAC      3673
TaABCB1-D      TGCGGCTCCTTCAGCGCCATCTTCGCCTACATCCTCAGCGCGGTGCTCAGCATCTACTAC      3688
               ***************** ******** *********************************

TaABCB1-A      ACGCCGGACCCGAGGCACATGGACCGGGAGATCGCCAAGTACTGCTACCTCCTCATCGGC      4631
TaABCB1-B      ACGCCGGACCCGAGGCACATGGACCGGGAGATCACCAAGTACTGCTACCTCCTCATCGGC      3733
TaABCB1-D      ACGCCGGACCCGAGGCACATGGACCGGGAGATCGCCAAGTACTGCTACCTCCTCATCGGC      3748
               ******************************** ***************************

TaABCB1-A      ATGTCCTCTGCCGCGCTGCTCTTCAACACCGTGCAGCACCTCTTCTGGGACACGGTGGGC      4691
TaABCB1-B      ATGTCCTCCGCCGCGCTGCTCTTCAACACCGTGCAGCACCTGTTCTGGGACACGGTGGGC      3793
TaABCB1-D      ATGTCCTCCGCCGCGCTGCTCTTCAACACCGTGCAGCACCTCTTCTGGGACACGGTGGGC      3808
               ******** ******************************** ******************
```

```
TaABCB1-A     GAGAACCTCACCAAGCGCGTGCGCGAGAAGATGCTCACGGCGGTGCTCCGCAACGAGATG     4751
TaABCB1-B     GAGAACCTCACCAAGCGCGTGCGCGAGAAGATGCTCACGGCGGTGCTCCGCAACGAGATG     3853
TaABCB1-D     GAGAACCTCACCAAGCGCGTGCGCGAGAAGATGCTCACGGCGGTGCTCCGCAACGAGATG     3868
              ************************************************************

TaABCB1-A     GCCTGGTTCGACATGGAGGCCAATGCCAGCGCGCACATCGCTGCCAGGCTGGCGCTGGAC     4811
TaABCB1-B     GCCTGGTTCGACATGGAGGCCAATGCGAGTGCGCACATCGCTGCCAGGCTCGCGCTGGAC     3913
TaABCB1-D     GCCTGGTTCGACATGGAGGCCAATGCCAGCGCGCACATCGCTGCCAGGCTCGCGCTGGAC     3928
              ************************** ** ******************** *********

TaABCB1-A     GCCCAGAACGTGCGCTCCGCCATCGGGGACCGCATCTCCATCATCGTGCAGAACTCGGCG     4871
TaABCB1-B     GCCCAGAACGTGCGCTCCGCCATCGGGGACCGCATCTCCATCATCGTGCAGAACTCGGCG     3973
TaABCB1-D     GCCCAGAACGTGCGCTCCGCCATCGGGGACCGCATCTCCATCATCGTGCAGAACTCGGCG     3988
              ************************************************************

TaABCB1-A     CTTATGCTCGTCGCATGCACGGCCGGGTTCGTCCTGCAGTGGCGCCTCGCGCTCGTGCTC     4931
TaABCB1-B     CTTATGCTCGTCGCATGCACGGCCGGGTTCGTCCTGCAGTGGCGCCTCGCGCTCGTGCTC     4033
TaABCB1-D     CTTATGCTCGTCGCCTGCACGGCCGGGTTCGTCCTGCAGTGGCGCCTCGCGCTCGTGCTC     4048
              ************** *********************************************

TaABCB1-A     CTCGCCGTCTTCCCACTCGTCGTCGGCGCCACCGTCCTGCAGAAGATGTTCATGAAGGGT     4991
TaABCB1-B     CTCGCCGTCTTCCCACTCGTCGTCGGCGCCACCGTCCTGCAGAAGATGTTCATGAAGGGT     4093
TaABCB1-D     CTCGCCGTCTTCCCACTCGTCGTCGGCGCCACCGTCCTGCAGAAGATGTTCATGAAGGGT     4108
              ************************************************************

TaABCB1-A     TTCTCGGGTGACCTGGAAGGCGCGCACGCCAAGGCGACGCAGATTGCGGGGGAGGCGGTG     5051
TaABCB1-B     TTCTCGGGTGACCTGGAAGGCGCGCACGCCAAGGCGACGCAGATTGCGGGGGAGGCTGTG     4153
TaABCB1-D     TTCTCGGGTGACCTGGAAGCCGCGCACGCCAAGGCGACGCAAATTGCGGGGGAGGCGGTG     4168
              ******************* ********************* ************** ***

TaABCB1-A     GCCAACGTGCGCACCGTGGCGGCGTTCAACTCGGAGGACAAGATCACGAGGCTCTTCGAG     5111
TaABCB1-B     GCCAACGTGCGCACCGTGGCGGCGTTCAACTCGGAGGACAAGATCACGAGGCTCTTCGAG     4213
TaABCB1-D     GCCAACGTGCGCACCGTGGCGGCGTTCAACTCGGAGGACAAGATCACGAGGCTCTTCGAG     4228
              ************************************************************

TaABCB1-A     GCCAACCTGCAGAGGCCGCTCCGTCGCTGCTTCTGGAAGGGCCAGATCGCGGGCATCGGC     5171
TaABCB1-B     GCCAACCTGCAGAGGCCGCTCCGGCGCTGCTTCTGGAAGGGCCAGATCGCCGGCATCGGC     4273
TaABCB1-D     GCCAACCTGCAGAGGCCGCTCCGGCGCTGCTTCTGGAAGGGCCAGATCGCCGGCATCGGC     4288
              *********************** ************************** *********

TaABCB1-A     TACGGTGTGGCGCAGTTCCTGCTGTACGCGTCCTACGCGCTGGGCCTGTGGTACGCTGCG     5231
TaABCB1-B     TACGGTGTGGCGCAGTTCCTGCTGTACGCGTCCTACGCGCTGGGCCTGTGGTACGCTGCG     4333
TaABCB1-D     TACGGTGTGGCGCAGTTCCTGCTGTACGCGTCCTACGCGCTGGGCCTGTGGTACGCTGCG     4348
              ************************************************************

TaABCB1-A     TGGCTGGTGAAGCACGGCATCTCCGACTTCTCCAAGACCATCCGCGTCTTCATGGTGCTC     5291
TaABCB1-B     TGGCTGGTGAAGCACGGCATCTCCGACTTCTCCAAGACCATCCGCGTCTTCATGGTGCTC     4393
TaABCB1-D     TGGCTGGTGAAGCACGGCGTCTCCGACTTCTCCAAGACCATCCGCGTCTTCATGGTGCTC     4408
              ****************** *****************************************

TaABCB1-A     ATGGTCTCCGCCAACGGTGCCGCCGAGACGCTGACGCTGGCGCCGGACTTCATCAAGGGC     5351
TaABCB1-B     ATGGTCTCCGCCAACGGTGCCGCTGAGACGCTGACGCTGGCGCCGGACTTCATCAAGGGC     4453
TaABCB1-D     ATGGTCTCCGCCAACGGTGCCGCCGAGACGCTGACGCTGGCGCCGGACTTCATCAAGGGC     4468
              *********************** ************************************

TaABCB1-A     GGGCGGGCCATGCAGTCGGTGTTCGAGACCATCGACCGCAAGACGGAGATCGAGCCCGAC     5411
TaABCB1-B     GGGCGGGCCATGCAGTCGGTGTTCGAGACCATCGACCGCAAGACGGAGATCGAGCCCGAC     4513
TaABCB1-D     GGGCGGGCCATGCAGTCGGTGTTCGAGACCATCGACCGCAAGACGGAGATCGAGCCCGAC     4528
              ************************************************************

TaABCB1-A     GACGTGGACGCCGCAGCAGTCCCCGAGCGTCCCAGGGGCGACGTGGAGTTGAAGCACGTC     5471
TaABCB1-B     GACGTGGACGCCGCAGCCGTCCCCGAGCGGCCCAGGGGCGACGTGGAGTTGAAGCACGTC     4573
TaABCB1-D     GACGTGGACGCCGCAGCCGTCCCCGAGCGGCCCAGGGGCGACGTGGAGTTGAAGCACGTC     4588
              ***************** *********** ******************************

TaABCB1-A     GACTTCTCGTACCCGTCGCGGCCGGACGTTCAGGTGTTCCGGGACCTGAGCCTCCGCGCC     5531
TaABCB1-B     GACTTCTCGTACCCGTCGCGGCCGGACATTCAGGTGTTCCGGGACCTGAGCCTCCGCGCG     4633
TaABCB1-D     GACTTCTCGTACCCGTCGCGGCCGGACGTTCAGGTGTTCCGGGACCTGAGCCTCCGCGCC     4648
              *************************** *******************************

TaABCB1-A     CGCGCAGGCCGGACGCTGGCGCTGGTGGGGCCCAGCGGGTGCGGCAAGAGCTCCGTGCTG     5591
TaABCB1-B     CGCGCAGGCCGGACGCTGGCGCTGGTGGGGCCCAGCGGGTGCGGCAAGAGCTCCGTGCTG     4693
TaABCB1-D     CGCGCAGGCCGGACGCTGGCGCTGGTGGGGCCCAGCGGGTGCGGCAAGAGCTCCGTGCTG     4708
              ************************************************************

TaABCB1-A     GCGCTCATCCAGCGCTTCTACGAGGCCAGCTCCGGGCGCGTGCTCCTGGACGGCAAGGAC     5651
TaABCB1-B     GCGCTCATCCAGCGCTTCTACGAGCCCAGCTCCGGGCGCGTGCTCCTGGACGGCAAGGAC     4753
TaABCB1-D     GCGCTCATCCAGCGCTTCTACGAGCCCAGCTCCGGGCGCGTGCTCCTGGACGGCAAGGAC     4768
              ************************ ***********************************
```

```
TaABCB1-A      ATCCGCAAGTACAACCTCAAGGCGCTGCGGCGAGTGGTGGCCATGGTGCCGCAGGAGCCG      5711
TaABCB1-B      ATCCGCAAGTACAACCTCAAGGCGCTGCGGCGAGTGGTGGCCATGGTGCCGCAGGAGCCG      4813
TaABCB1-D      ATCCGCAAGTACAACCTCAAGGCGCTGCGGCGAGTGGTGGCCATGGTGCCGCAGGAGCCG      4828
               ************************************************************

TaABCB1-A      TTTCTTTTCGCCGGCACCATCCACGACAACATCGCCTACGGGCGCGAGGGCGCGACCGAG      5771
TaABCB1-B      TTTCTTTTCGCCGGCACCATCCACGACAACATCGCCTACGGGCGCGAGGGCGCGACCGAG      4873
TaABCB1-D      TTTCTTTTCGCCGGCACCATCCACGACAACATCGCCTACGGCCGCGAGGGCGCTACCGAG      4888
               ***************************************** *********** ******

TaABCB1-A      GCGGAGGTGGTGGAGGCGGCAACGCAGGCCAACGCGCACAAGTTCGTGTCGGCGCTGCCG      5831
TaABCB1-B      GCGGAGGTGGTGGAGGCGGCAACGCAGGCCAACGCGCACAAGTTCGTGTCGGCGCTGCCG      4933
TaABCB1-D      GCGGAGGTGGTGGAGGCGGCAACGCAGGCCAACGCGCACAAGTTCGTGTCGGCGCTGCCG      4948
               ************************************************************

TaABCB1-A      GAAGGGTACAAGACGTGCGTCGGGGAGCGCGGGGTGCAGCTGTCGGGAGGGCAGCGCCAG      5891
TaABCB1-B      GAAGGGTACAAGACGTGCGTCGGGGAGCGCGGGGTGCAGCTGTCGGGAGGGCAGCGGCAG      4993
TaABCB1-D      GAAGGGTACAAGACGTGCGTCGGGGAGCGCGGGGTGCAGCTGTCGGGAGGGCAGCGCCAG      5008
               ******************************************************** ***

TaABCB1-A      CGGATCGCCATCGCGCGAGCGCTGGTGAAGCAGGCGGCCATCATGCTGCTGGACGAGGCG      5951
TaABCB1-B      CGGATCGCCATCGCGCGAGCGCTGGTGAAGCAGGCGGCCATCATGCTGCTGGACGAGGCG      5053
TaABCB1-D      CGGATCGCCATCGCGCGAGCGCTGGTGAAGCAGGCGGCCATCATGCTGCTGGACGAGGCG      5068
               ************************************************************

TaABCB1-A      ACGAGTGCGCTGGACGCCGAGTCTGAGCGGTGCGTGCAGGAGGCGCTGGACCGGGCCGGG      6011
TaABCB1-B      ACGAGTGCCCTGGACGCCGAGTCGGAGCGGTGCGTGCAGGAGGCGCTGGACCGGGCCGGG      5113
TaABCB1-D      ACGAGTGCGCTGGACGCCGAGTCGGAGCGGTGCGTGCAGGAGGCGCTGGACCGGGCCGGG      5128
               ******** ************** ************************************

TaABCB1-A      CCAGGGTCAGGGCGAACGACCATCGTGGTGGCGCACCGCCTGGCCACGGTGCGGAACGCG      6071
TaABCB1-B      TCAGGGTCAGGGCGAACGACCATCGTGGTGGCGCACCGGCTGGCCACGGTGCGGAACGCG      5173
TaABCB1-D      TCAGGGTCAGGGCGAACGACCATCGTGGTGGCGCACCGCCTGGCCACGGTGCGGAACGCG      5188
                ************************************* *********************

TaABCB1-A      CACACCATCGCGGTCATCGACGACGGCAAGGTGGTGGAGCAAGGGTCGCACTCGCACCTG      6131
TaABCB1-B      CACACCATCGCGGTGATCGACGACGGCAAGGTGGTGGAGCAAGGGTCGCACTCGCATCTG      5233
TaABCB1-D      CACACCATCGCGGTCATCGACGACGGCAAGGTGGTGGAGCAAGGGTCGCACTCGCACCTG      5248
               ************** ***************************************** ***

TaABCB1-A      CTCAACCATCACCCCGACGGATGCTACGCGCGGATGCTGCAGCTGCAGCGCCTCACGCCC      6191
TaABCB1-B      CTCAACCATCACCCCGACGGATGCTACGCGCGGATGCTGCAGTTCCAGCGCCTCACGCCC      5293
TaABCB1-D      CTCAACCATCACCCCGACGGATGCTACGCGCGGATGCTGCAGCTGCAGCGCCTCACGCCC      5308
               ****************************************** * ***************

TaABCB1-A      CACACCCTTGCCGTGCCCGGACCCGGACCCGGATCCTCTGCATCCAACGTTTGA            6245
TaABCB1-B      CACACCCTTGCCGTGCCCGGACCCTCT------------GCATCCAACGTTTGA            5335
TaABCB1-D      CACACCCTTGCCGTGCCCGGACCCGGACCCTCT------GCATCCAACGTTTGA            5356
               ************************    *            *************
```

Supplementary Fig. S2. Sequence comparison of homoeologous copies from translation start to stop site. All sequences are from 5' to 3' direction.

A

```
TaABCB1-B      ------------------------------------------------------------   0
TaABCB1-A      ------------------------------------------------------------   0
TaABCB1-D      CTGCCCGCCGATGAATGTTAATATCAACAGCCAGACTACAACAAATGATACTACCTAGCT   60

TaABCB1-B      ------------------------------------------------------------   0
TaABCB1-A      ------------------------------------------------------------   0
TaABCB1-D      GGCTGTATCTGTCCTTAGTGGATCACTATTGTGAAGAGGAGGAGATTTGCTCAAGAGCAA   120

TaABCB1-B      ------------------------------------------------------------   0
TaABCB1-A      ------------------------------------------------------------   0
TaABCB1-D      GAGAGTGGTAGGAAGGATGTCGAGCGTGTTTGGTGTTCTGCAACCTCGATGGGGCATCGT   180

TaABCB1-B      -----GGCAAGCTGGCCGTGCGGGCAAATACGAGTCACCGCATTGACACACTACCAACCC   55
TaABCB1-A      ------------------------------------------------CACGGCTACCTC   12
TaABCB1-D      TCGGTATCCTGCTAGAATTTGGAGCACCAAGGAGTTGTGGGAGGTGATGACTGCTTGTGT   240
                                                                **  *

TaABCB1-B      AAACTAAAAACAGGACGGACGCCAAACGCTCCGCCGGCATAAACGAACAAAAAA----TA   111
TaABCB1-A      GACCACGG----CTCCACCAC---------CCTACGCTCTCGGCTACCTCAACATCGGCA   59
TaABCB1-D      GATCATGA----ATGAGCTCCCGAAACGTATCTACGATCAAGGGTTTCAGTTCCAGGGTG   296
                * *                           *  **           *

TaABCB1-B      GACAAAGCGTGCATCGGTTTGGGTCGGCGGCGC-GTTGAAGCTGC---TCTAATGTGCAC   167
TaABCB1-A      CAAAGGGTTATCATCCGCTTGAG------------------CAACT-AGTCGGCTTCGTC   100
TaABCB1-D      ACAATGTTGTGCCTGAGCATGGAGGAGCGGCAACGTTTGCACAGTTTATCCAATTTCATC   356
                  *       * *  *  **                    *             *   *

TaABCB1-B      AGCACACCTCTCGAAAAGTCATTTGAGGGCACCACATCGGCACCGGTAG------GAAGA   221
TaABCB1-A      TACCGTCAACGCGTCCGCAACGCGACACCGTCTGCGACGCTCCCGCTAC--GACTGCGGG   158
TaABCB1-D      ATTAATTTTGTGATTAGGAAACTCACGGGCTCATGTTGGCAATCAATAGATGTAGTTTTT   416
                                              *      *    *  **

TaABCB1-B      GGGTGTTTGTTTCCAGGGTCTTATTGGTGTAGGGACTT------AAAAAAGTCTTTGTAA   275
TaABCB1-A      GGGGTGTCAGCCCGCCGGCTGCTATTCTCTCCAGTCTGACCGTCCGCGACGGTCATGTTG   218
TaABCB1-D      AAAATGTAATTAAGACCATTTAATTTTTGTTCAGCTTGTAAACTAGATATATTTATTTAG   476
                     *                 *  * *   *  *           *      * *

TaABCB1-B      GTCC--CATCTAAACCAAACAGGA-GAAATTTATAAGGACTTAAAATGAACATTTTGAAC   332
TaABCB1-A      TTCGCCACGC---------TACCGCTACGACTGCGGGGATGTTAGATAGAGTTATTTCCA   269
TaABCB1-D      GTTGTGGAACTATGCTATATTTGTCTGCTTATGAAACTATGCAAAATATGTGTATATTGG   536
                *       *                     *      *    * **     * *

TaABCB1-B      TTATGAAATAAGACTCTTAAGGAGAGTTTTATAAGGACTTATAGTTGTAATATG------   386
TaABCB1-A      TCCTATCTCTAGAA-----GGTGTCTTGCCCTCAAGTCTTGTACTCAATATATAGTCGGC   324
TaABCB1-D      TTG--AAAAATGAC-----AGCAAGCTGGCCGTACGGCAAATATGAGTTATCGCGTTGGA   589
               *          **       *     *      * * *   **      **

TaABCB1-B      ------GTCTTTTAGTCCATGTTAAATCCCAGGAACCAAACAAGTAGTGACTTTTTAGGG   440
TaABCB1-A      TTCGAGCCTCAATAATCCATCATATTC--CGCAAATCTCCCCACTCTTCCTTGGTGGACG   382
TaABCB1-D      CGCATTGTCGACCCAAACAAATAAAAA--CCGGACAAAACGCACATCCGTTTGGTGGACG   647
                                **    *     *   *        *        *  *    *

TaABCB1-B      ACTT---------------AAAACTTATAAGTTGGGACAAAAAAGTCCTAAGACTTATGA   485
TaABCB1-A      CATTGAAGTTGCTCTAACGTGCACTGCACGCCTCTCGAGAAGTCATTTGAGGGCACCACA   442
TaABCB1-D      TATTGAAGTTGCTCTAACGTGCACTGCACGCCTCTCGAGAAGTCATTTGAGGGCACCACA   707
                 **                  ***       *      **    *   * * *     *

TaABCB1-B      ACCAAACAGGGCCGAACTCACGAAAGAAGAACCACTGCACTGTACGGGGAAGTGTACCGC   545
TaABCB1-A      CCGGTACTGGTAGGAACTCAGGAAAGAATAACCAGTGCACTGTACGGGGAAGTGTGCCGC   502
TaABCB1-D      CCGGCGCTGGTAGGAACTCACGAAAGAAGAACCACTGCACCGTGTGAGC-----------   756
                *    * **   ******* ******* ***** ***** **  * *

TaABCB1-B      GAGCATCTTCGTTGACAAAGTCG-TGGGAAAAAGGAATGTGCGCTCAACATGCAGTTTCCA   604
TaABCB1-A      GAGCATCTTCATTGAGAAAGTCGTGGGAAAAAGGAAAGTGCGCTCAACATGCAGTTTCCA   562
TaABCB1-D      ----ATCTTCATTGACAAAGTCG-TGGGAAAAGGAAAGTGCGCTCAACATGCAGTTTCCA   811
                   ****** **** *******  ** ******** ***********************

TaABCB1-B      CTTACTCACGATTATGGACGTAAACAAATTACTACAGAGATGGGATTTGATTCTTTTGCT   664
TaABCB1-A      CTGACTCACGATTATGGACGTAAACAAATTACTACAGAGATGGGATTTGATTCTTTTGCT   622
TaABCB1-D      CTTACTCACGATTATGGACGTAAACAAATTACTACAGAGATGGGATTTGATTCTTTTGCT   871
               ** *********************************************************

TaABCB1-B      AAAGAAACATTATAGATAGGAGTGTGA--------------------GTGTGTGCATTTT   704
TaABCB1-A      AAAGAAACATTATGGATAGGAATGTGAGTGTATGTATGTCTTTGTGAGTATGTGCATTTT   682
TaABCB1-D      AAAGAAACATTATGGATAGGAATGTGAGTGTATGTATGTGTTTGTGAGTATGTGCATTTT   931
               ************* ******* *****                    ** **********
```

B

```
TaABCB1-B       TGGTGGAGCAAGGGTCGCACTCGCATCTGCTCAACCATCACCCCGACGGATGCTACGCGC 60
TaABCB1-A       TGGTGGAGCAAGGGTCGCACTCGCACCTGCTCAACCATCACCCCGACGGATGCTACGCGC 60
TaABCB1-D       TGGTGGAGCAAGGGTCGCACTCGCACCTGCTCAACCATCACCCCGACGGATGCTACGCGC 60
                ************************* **********************************

TaABCB1-B       GGATGCTGCAGTTCCAGCGCCTCACGCCCCACACCCTTGCCGTGCCCGGACCC------- 113
TaABCB1-A       GGATGCTGCAGCTGCAGCGCCTCACGCCCCACACCCTTGCCGTGCCCGGACCCGGACCCG 120
TaABCB1-D       GGATGCTGCAGCTGCAGCGCCTCACGCCCCACACCCTTGCCGTGCCCGGACCCGGAC--- 117
                *********** * ********************* *****************

TaABCB1-B       -----TCTGCATCCAACGTTTGATACATCTTCCC-------TTCCATGCACGGTTCCACT 161
TaABCB1-A       GATCCTCTGCATCCAACGTTTGATTCATCTTCCACGCACGGTTCCACTCGCG-TTTTGCT 179
TaABCB1-D       ---CCTCTGCATCCAACGTTTGATACATCTTCCATGCACGGTTCCACTCGCG-TTTTGCT 173
                     ******************* ********        *****  * ** **   **

TaABCB1-B       TTAGGATCTTCTTTTCTTCTAAAAAAT----------TTGTTGCTGGCGGGAGG------ 205
TaABCB1-A       TTAGGATCTTCTTTTCTTCTAAAAAATTTGCGAAAATTTGTTGCTGGCTGGCGGATTGGT 239
TaABCB1-B       TTAGGATCTTCTTTTCTTCTAAAAAAT----------TTGTTGCTGGCTGGCGGGA---- 219
                ***************************          *********** ** **

TaABCB1-B       ---AGGGTGGTCACGATTGACAAGCCGTATGTGCTGCCAATGCCATGATCCG-------- 254
TaABCB1-A       TGGAGGGTGGTCACAATTGACAAGCCGTATGTGCTACCAATGCCATGATCCGTATTACAC 299
TaABCB1-D       -GGAGGGTGGTCACAATTGACAAGCCGTATGTGCTGCCAATGCCATGATCCG-------- 270
                   *********** ******************** ****************
```

Supplementary Fig. S3: A) Sequence comparison of untranslated region upstream of translation start site of homoeologous copies; B) untranslated region downstream of translation stop site. Allsequences are from 5’ to 3’ direction.

```
                                                   Monocot specific
TaABCB1-A   MSSDPEEIKARVVVHG--ADA--AD-EWARPELEAFHLPSTSQPPHLFHPPHPEPEAAEQS   56
TaABCB1-B   MSSDPEEIKARVVVHGADADAD-ADAEWARPELEAFHLPSTSQPPHLFHPPHPEPEAAEQS   60
TaABCB1-D   MSSDPEEIKARVVVHGAGADANAAD-EWARPELEAFHLPSTSQPPHLFHPPHPEPEAAEQS   60
            ****************  ***  ** **********************************

TaABCB1-A   TPAPAVAAAT-TNNASGSPPP----PPAPLETEQLPPNAKPPADEKPPPPAPAAALRDLF  111
TaABCB1-B   TPAPAVAAAT-TNNASGSPPPPRSPPPAPLETEQLPPNAKPPADEKPPPPAPAAALRDLF  119
TaABCB1-D   TPAPAVAAATGTNNASGSPPPPRSPPPAPLETEQLPPNAKPPADEKPPPPAPAAALRDLF  120
            ********** **********    ***********************************

TaABCB1-A   RFADGLDRVLMAVGTLGALVHGCSLPVFLRFFADLVDSFGSHADDPDTMVRLVVKYAFYF  171
TaABCB1-B   RFADGLDRVLMAVGTLGALVHGCSLPVFLRFFADLVDSFGSHADDPDTMVRLVVRYAFYF  179
TaABCB1-D   RFADGLDRVLMAVGTLGALVHGCSLPVFLRFFADLVDSFGSHADDPDTMVRLVVKYAFYF  180
            ******************************************************:*****
                                                        UM1
TaABCB1-A   LVVGAAIWASSWAEISCWMWTGERQSTRMRIRYLQAALKQDVSFFDTDVRTSDVIYAINA  231
TaABCB1-B   LVVGAAIWASSWAEISCWMWTGERQSTRMRIRYLQAALKQDVSFFDTDVRTSDVIYAINA  239
TaABCB1-D   LVVGAAIWASSWAEISCWMWTGERQSTRMRIRYLQAALKQDVSFFDTDVRTSDVIYAINA  240
            ************************************************************

TaABCB1-A   DAVMVQDAISEKLGNLIHYMATFVAGFVVGFTAAWQLALVTLAVVPLIAVIGGLTAATMG  291
TaABCB1-B   DAVIVQDAISEKLGNLIHYMATFVAGFVVGFTAAWQLALVTLAVVPLIAVIGGLTAATMG  299
TaABCB1-D   DAVIVQDAISEKLGNLIHYMATFVAGFVVGFTAAWQLALVTLAVVPLIAVIGGLTAATMG  300
            ***:********************************************************
                         Transmembrane Domain 1
TaABCB1-A   KLSSKSQDALSSASNIAEQALSQIRVVQSFVGEERVAQAYSAALAVAQSIGYRNGFAKGL  351
TaABCB1-B   KLSSKSQDALSSASNIAEQALSQIRIVQSFVGEQRVAQAYSAALAVAQSIGYRNGFAKGL  359
TaABCB1-D   KLSSKSQDALSSASNIAEQALSQIRIVQSFVGEQRVAQAYSEALAVAQSIGYRNGFAKGL  360
            *************************:*******:******* ******************

TaABCB1-A   GLGGTYFTVFCCYALLLWYGGHLVRGHHTNGGLAIATMFSVMIGGLGLGQSAPSMAAFAK  411
TaABCB1-B   GLGGTYFTVFCCYALLLWYGGHLVRGHHTNGGLAIATMFSVMIGGLALGQSAPSMAAFAK  419
TaABCB1-D   GLGGTYFTVFCCYALLLWYGGHLVRGHHTNGGLAIATMFSVMIGGLGLGQSAPSMAAFAK  420
            **********************************************.*************
                                                UM2
TaABCB1-A   ARVAAAKIFRIIDHTPGITKEGVELESVTGRLELRNVEFAYPSRPDTPILRRFSLSVPAG  471
TaABCB1-B   ARVAAAKIFRIIDHTPGVTKEGVELESVTGRLELRNVEFAYPSRPDTPILRRFSLSVPAG  479
TaABCB1-D   ARVAAAKIFRIIDHTPGITKEGVELESVTGRLELRNVEFAYPSRPDTPILRRFSLSVPAG  480
            *****************:******************************************
               Walker A     UM3                                 Q-loop
TaABCB1-A   KTIALVGSSGSGKSTVVSLIERFYDPSSGQIMLDGVELKDLKLRWLRSQIGLVSQEPALF  531
TaABCB1-B   KTIALVGSSGSGKSTVVSLIERFYDPSSGQIMLDGVELKDLKLRWLRSQIGLVSQEPALF  539
TaABCB1-D   KTIALVGSSGSGKSTVVSLIERFYDPSSGQIMLDGVELKDLKLRWLRSQIGLVSQEPALF  540
            ************************************************************
                                                  ABC transport signature
TaABCB1-A   ATSIRENLLLGREEASQVEMEEAARVANAHSFIIKLPDGYDTQVGERGLQLSGGQKQRIA  591
TaABCB1-B   ATSIRENLLLGREEASQVEMEEAARVANAHSFIIKLPDGYDTQVGERGLQLSGGQRQRIA  599
TaABCB1-D   ATSIRENLLLGREEASQVEMEEAARVANAHSFIIKLPDGYDTQVGERGLQLSGGQKQRIA  600
            *******************************************************:****
                Walker B D-loop                         H-loop
TaABCB1-A   IARAMLKNPAILLLDEATSALDSESEKLVQEALDRFMIGRTTLVIAHRLSTIRKADLVAV  651
TaABCB1-B   IARAMLKNPAILLLDEATSALDSESEKLVQEALDRFMIGRTTLVIAHRLSTIRKADLVAV  659
TaABCB1-D   IARAMLKNPAILLLDEATSALDSESEKLVQEALDRFMIGRTTLVIAHRLSTIRKADLVAV  660
            ************************************************************
                       Nucleotide Binding Domain 1
TaABCB1-A   LQAGAVSEMGAHDDLMARGDSGAYAKLIRMQEQAHEAAIVSARRSSARPSSARNSVSSPI  711
TaABCB1-B   LQAGAVSEMGAHDDLMARGDSGAYAKLIRMQEQAHEAALVSARRSSARPSSARNSVSSPI  719
TaABCB1-D   LQAGAVSEMGAHDDLMARGDSGAYAKLIRMQEQAHEAALVSARRSSARPSSARNSVSSPI  720
            **************************************:*********************

TaABCB1-A   MMRNSSYGRSPYSRRLSDFSTADFSLSVIHDPAAHRMGMG--MGMEKLAFRAQASSFWRL  769
TaABCB1-B   MMRNSSYGRSLYSRRLSDFSTADFSLSVIHDPAAHRMG----MGMEKLAFRAQASSFWRL  775
TaABCB1-D   MMRNSSYGRSPYSRRLSDFSTADFSLSVIHDPAAHRMGMGMGMGMEKLAFRAQASSFWRL  780
            ********** ***************************    ******************
                                 Linker region
TaABCB1-A   AKMNSPEWGYALAGSVGSMVCGSFSAIFAYILSAVLSIYYTPDPRHMDREIAKYCYLLIG  829
TaABCB1-B   AKMNSPEWGYALAGSVGSMVCGSFSAIFAYILSAVLSIYYTPDPRHMDREITKYCYLLIG  835
TaABCB1-D   AKMNSPEWGYALAGSVGSMVCGSFSAIFAYILSAVLSIYYTPDPRHMDREIAKYCYLLIG  840
            ***************************************************:********

TaABCB1-A   MSSAALLFNTVQHLFWDTVGENLTKRVREKMLTAVLRNEMAWFDMEANASAHIAARLALD  889
TaABCB1-B   MSSAALLFNTVQHLFWDTVGENLTKRVREKMLTAVLRNEMAWFDMEANASAHIAARLALD  895
TaABCB1-D   MSSAALLFNTVQHLFWDTVGENLTKRVREKMLTAVLRNEMAWFDMEANASAHIAARLALD  900
            ************************************************************

TaABCB1-A   AQNVRSAIGDRISIIVQNSALMLVACTAGFVLQWRLALVLLAVFPLVVGATVLQKMFMKG  949
TaABCB1-B   AQNVRSAIGDRISIIVQNSALMLVACTAGFVLQWRLALVLLAVFPLVVGATVLQKMFMKG  955
TaABCB1-D   AQNVRSAIGDRISIIVQNSALMLVACTAGFVLQWRLALVLLAVFPLVVGATVLQKMFMKG  960
            ************************************************************
                         Transmembrane Domain 2
TaABCB1-A   FSGDLEGAHAKATQIAGEAVANVRTVAAFNSEDKITRLFEANLQRPLRRCFWKGQIAGIG 1009
TaABCB1-B   FSGDLEGAHAKATQIAGEAVANVRTVAAFNSEDKITRLFEANLQRPLRRCFWKGQIAGIG 1015
TaABCB1-D   FSGDLEAAHAKATQIAGEAVANVRTVAAFNSEDKITRLFEANLQRPLRRCFWKGQIAGIG 1020
            ******.*****************************************************

TaABCB1-A   YGVAQFLLYASYALGLWYAAWLVKHGISDFSKTIRVFMVLMVSANGAAETLTLAPDFIKG 1069
TaABCB1-B   YGVAQFLLYASYALGLWYAAWLVKHGISDFSKTIRVFMVLMVSANGAAETLTLAPDFIKG 1075
TaABCB1-D   YGVAQFLLYASYALGLWYAAWLVKHGVSDFSKTIRVFMVLMVSANGAAETLTLAPDFIKG 1080
            **************************:*********************************

TaABCB1-A   GRAMQSVFETIDRKTEIEPDDVDAAAVPERPRGDVELKHVDFSYPSRPDVQVFRDLSLRA 1129
TaABCB1-B   GRAMQSVFETIDRKTEIEPDDVDAAAVPERPRGDVELKHVDFSYPSRPDIQVFRDLSLRA 1135
TaABCB1-D   GRAMQSVFETIDRKTEIEPDDVDAAAVPERPRGDVELKHVDFSYPSRPDVQVFRDLSLRA 1140
            *************************************************:**********
                 Walker A                                        Q-loop
TaABCB1-A   RAGRTLALVGPSGCGKSSVLALIQRFYEASSGRVLLDGKDIRKYNLKALRRVVAMVPQEP 1189
TaABCB1-B   RAGRTLALVGPSGCGKSSVLALIQRFYEPSSGRVLLDGKDIRKYNLKALRRVVAMVPQEP 1195
TaABCB1-D   RAGRTLALVGPSGCGKSSVLALIQRFYEPSSGRVLLDGKDIRKYNLKALRRVVAMVPQEP 1200
            ****************************.*******************************
                       Nucleotide Binding Domain 2        ABC transport signature
TaABCB1-A   FLFAGTIHDNIAYGREGATEAEVVEAATQANAHKFVSALPEGYKTCVGERGVQLSGGQRQ 1249
TaABCB1-B   FLFAGTIHDNIAYGREGATEAEVVEAATQANAHKFVSALPEGYKTCVGERGVQLSGGQRQ 1255
TaABCB1-D   FLFAGTIHDNIAYGREGATEAEVVEAATQANAHKFVSALPEGYKTCVGERGVQLSGGQRQ 1260
            ************************************************************
                   Walker B D-loop                           H-loop
TaABCB1-A   RIAIARALVKQAAIMLLDEATSALDAESERCVQEALDRAGPGSGRTTIVVAHRLATVRNA 1309
TaABCB1-B   RIAIARALVKQAAIMLLDEATSALDAESERCVQEALDRAGSGSGRTTIVVAHRLATVRNA 1315
TaABCB1-D   RIAIARALVKQAAIMLLDEATSALDAESERCVQEALDRAGSGSGRTTIVVAHRLATVRNA 1320
            *************************************.**********************

TaABCB1-A   HTIAVIDDGKVVEQGSHSHLLNHHPDGCYARMLQLQRLTPHTLAVPGPGPGSSASNV 1366
TaABCB1-B   HTIAVIDDGKVVEQGSHSHLLNHHPDGCYARMLQFQRLTPHTLAVPGP----SASNV 1368
TaABCB1-D   HTIAVIDDGKVVEQGSHSHLLNHHPDGCYARMLQLQRLTPHTLAVPGPGP--SASNV 1375
            **********************************:*************    *****
```

Supplementary Fig. S4: Amino acid sequence comparison and structural features among the three homoeologous copies. Transmembrane Domain (TMD) 1 and 2 and Nucleotide Binding Domain (NBD)1 and 2 are marked as bold lines below the sequence alignment. Motifs are marked above the alignment and differences between NBD1 and NBD2 are highlighted as bold. Mismatches present in 5', linker and 3' regions of gene among three copies are marked with boxes.

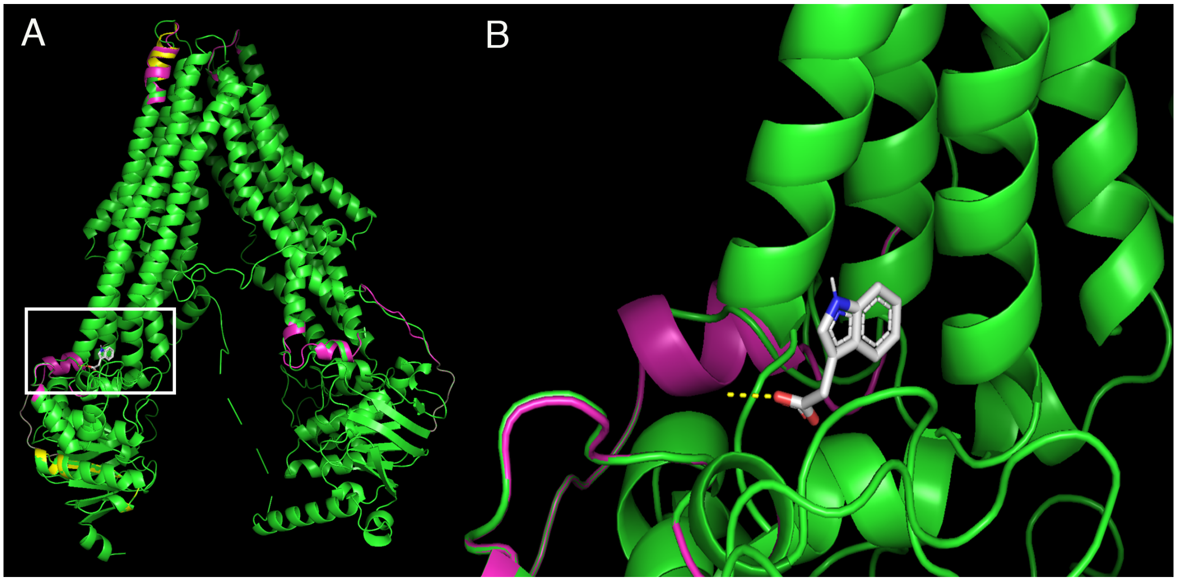

Supplementary Fig. S5. A) Molecular docking of natural auxin IAA molecule on the superimposed 3-D structure of TaABCB1 homoeologs (represented by superimposed green, pink and yellow color). B) Enlarged view of IAA molecule (white) docked on TaABCB1 protein.

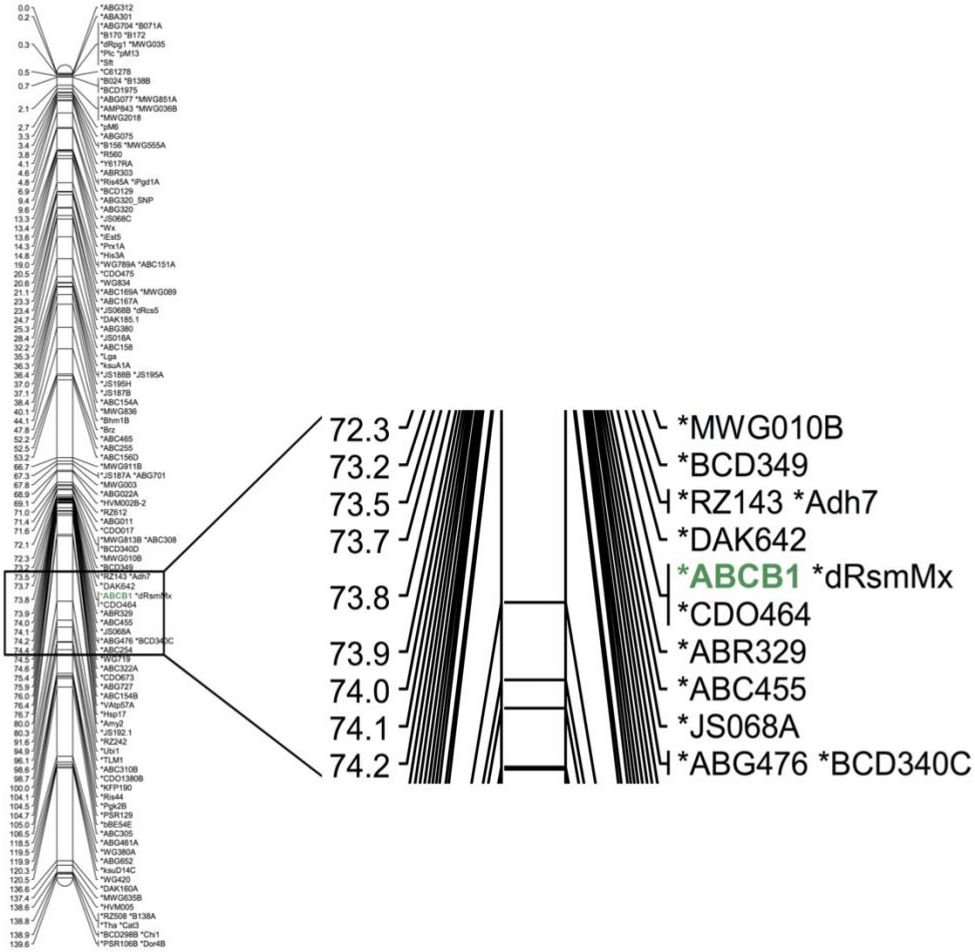

Supplementary Fig. S6: Genetic linkage map of barley showing location of *ABCB1* on chromosome 7HS near centromere. The distance among markers in cM was marked on the left of the chromosome.

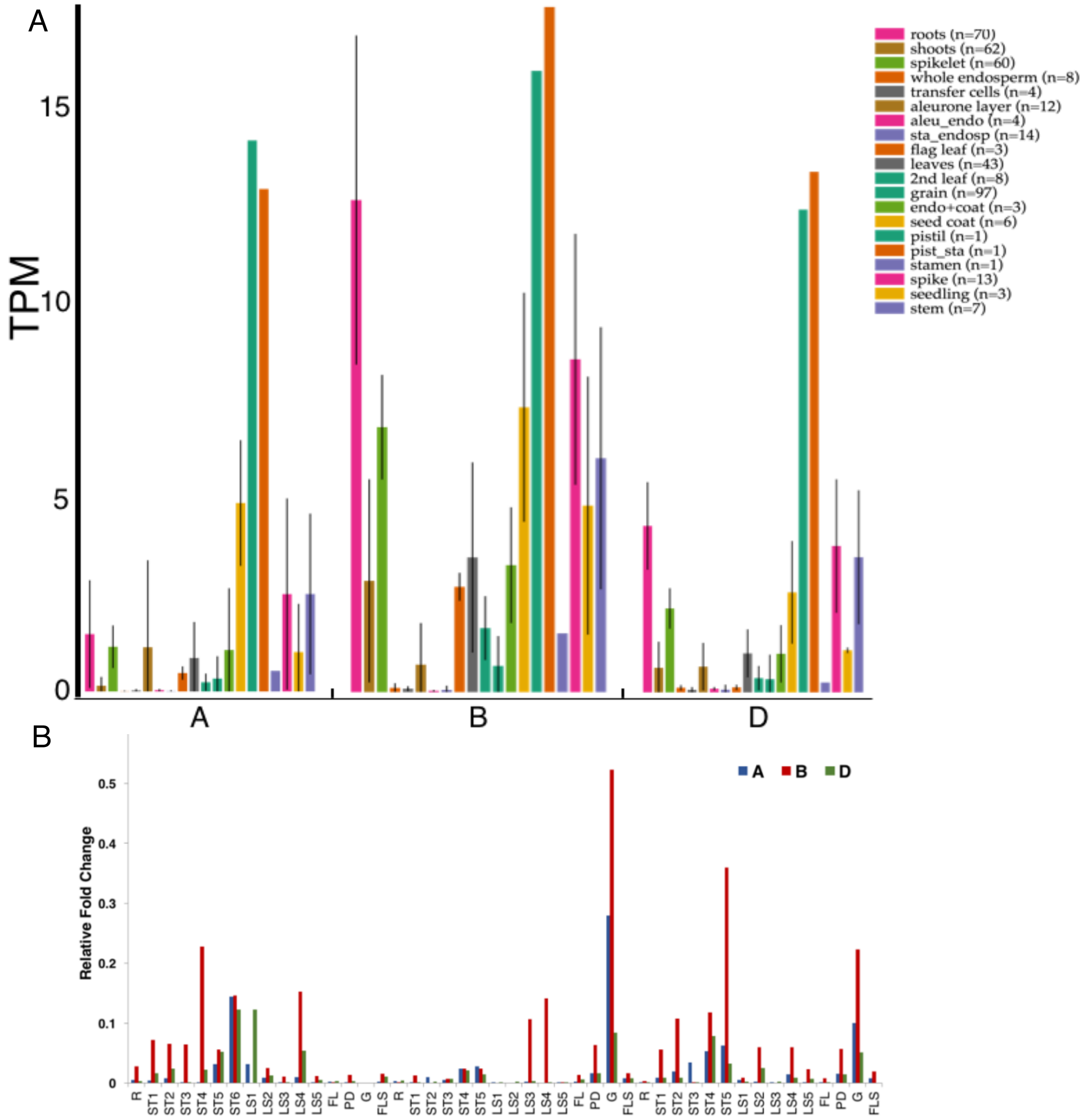

Supplementary Fig. S7: Expression of 7A, 7B and 7D homoeologs A) In-silico under normal condition in different tissues expressed as transcript per million. The number after the tissue indicates the number of samples in the study. The expression data set includes, more than 13 varieties, several NILs and group 1 and 5 Chinese spring nulli-tetra lines; B) RT-PCR analysis on three biological samples in cv. Sariab-92 on different tissues including nodes and internodes, leaf at different stages, pollen development and grain.

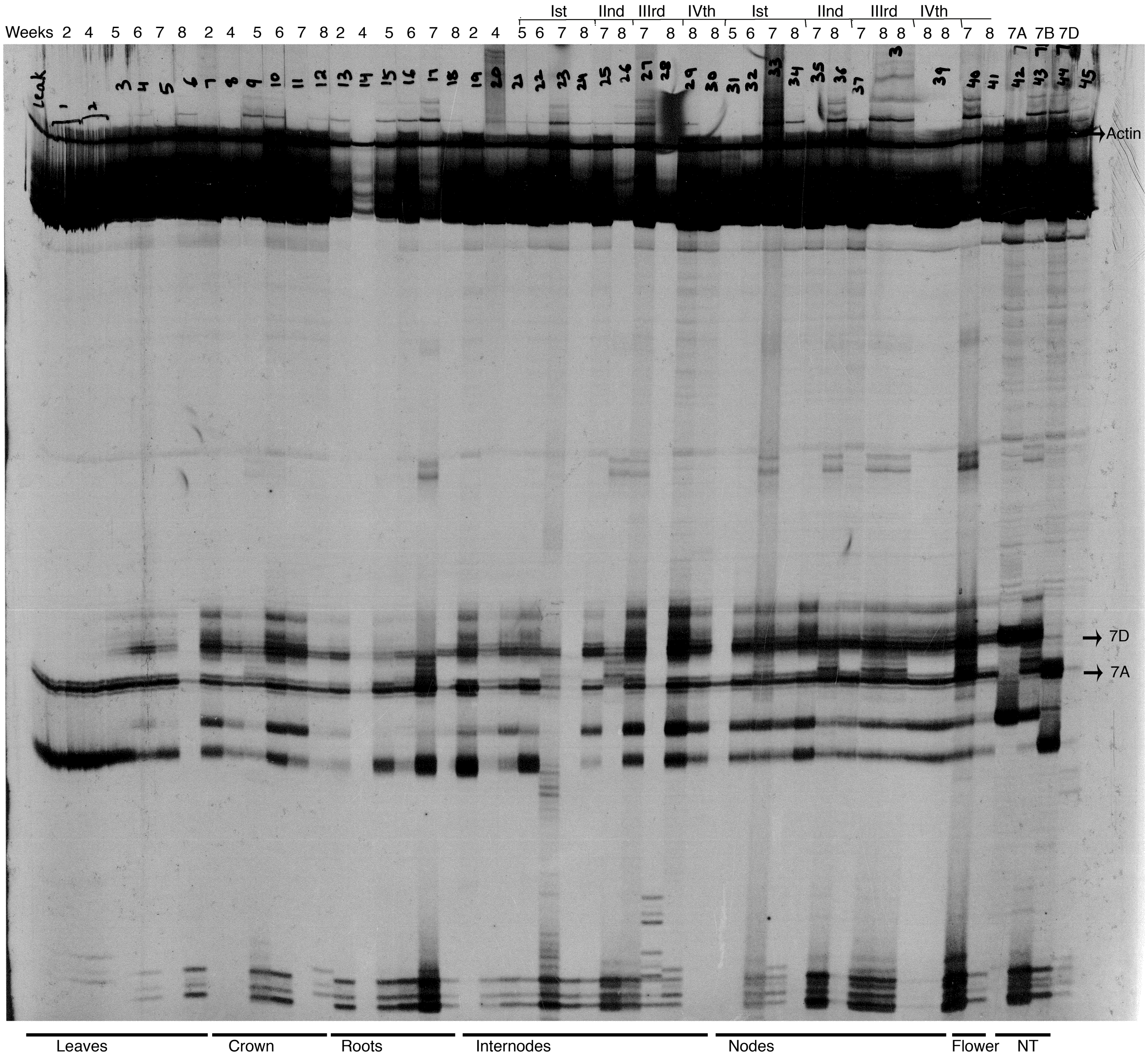

Supplementary Fig. S8: SSCP gel scanned X-ray showing expression of *TaABCB1* using common primer in different spatiotemporal tissues along with group 7 nulli-tetra lines. Top numbers show weeks after sowing and bottom represents tissue during respected weeks. Actin was used as an internal control and 7A and 7D on right represent respective copy band.

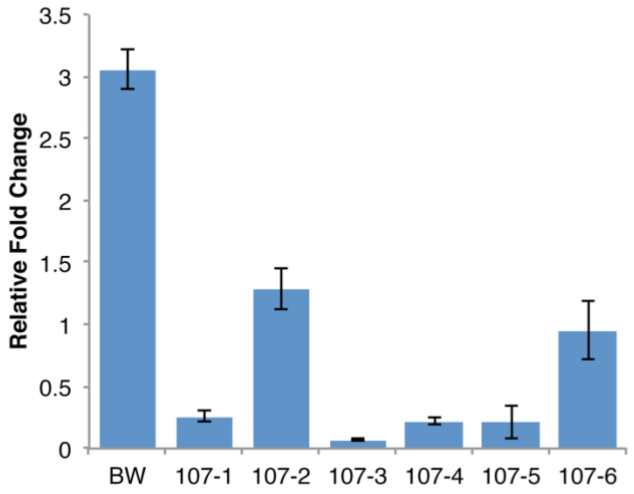

Supplementary Fig. S9: Expression of *TaABCB-1* gene from flag leaf of Bobwhite and TaABCB1 T2-107 plant 1 to 6.

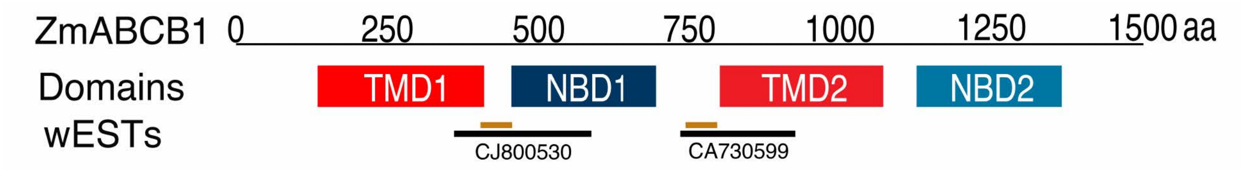

Supplementary Fig. S10: Predicted protein structure of the maize ABCB1 (ZmABCB1) gene showing the arrangement of the major domains. Alignment of the two orthologous wheat ESTs (black) and the unique regions (brown) used to develop oligos for VIGS, RNAi and mapping in wheat are marked below the protein structure.

Supplementary figures

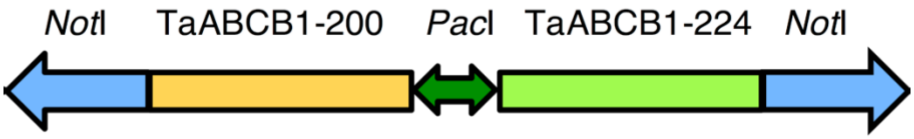

Supplementary Fig. S11: Design of oligo from wheat ESTs. Each fragment released by *Not*I or *Not*I and *Pac*I digestion was used for structural and functional analyses.

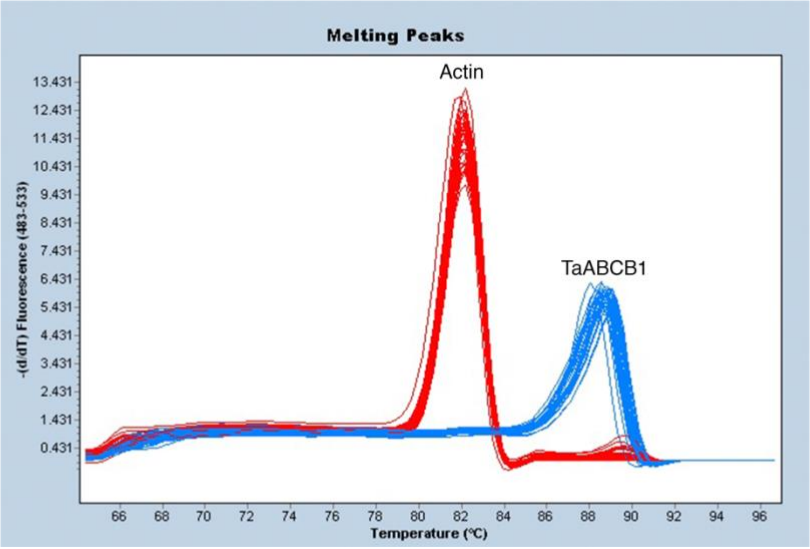

Supplementary Fig. S12: Representation figure for a melt curve analysis of internal control Actin (Act 247 F and R) and TaABCB1 common primer (TaABCB1-19F and R) using SYBR Green I chemistry on Roche LightCycler® 480 (Roche Diagnostics, USA).

Supplementary Table S1: Gene specific primer and oligo sequences used for cloning *TaABCB1* homoeologues, 5′ promoters regions, and 3′ UTRs, real-time quantitative PCR, SSCP analysis and RNAi analysis. Bases in italics letters represent *att*B overhangs.

**Primer/oligo (5′-3′)**	**Sequence 5′ to 3′**
TaABCB1-2F	CTTCAGCCTGTCCGTCATACA
TaABCB1-2R	ACTCGGGCGAGTTCATCTT
TaABCB1-4F	*GGGGACAAGTTTGTACAAAAAAGCAGGCT*ATGTCTAGCGACCCTGAAGAG
TaABCB1-4R	*GGGGACCACTTTGTACAAGAAAGCTGGGT*AACGTTGGATGCAGAGGGTC
TaABCB1-5F	*GGGGACAAGTTTGTACAAAAAAGCAGGCT*GCCAGAAGCAGCGGATC
TaABCB1-5R	*GGGGACCACTTTGTACAAGAAAGCTGGGT*CTGCACGAGCTTCTCCG
TAABCB1-6R	*GGGGACCACTTTGTACAAGAAAGCTGGGT*GGGTCGTGGAGGATGAATTG
TaABCB1-ApromoterF	*GGGGACAAGTTTGTACAAAAAAGCAGGCT*CATCGGCACAAAGGGTTATC
TaABCB1-ABpromoterR	*GGGGACCACTTTGTACAAGAAAGCTGGGT*GACGCATTGTTGGTGGTAGC
TaABCB1-BpromoterF	*GGGGACAAGTTTGTACAAAAAAGCAGGCT*AAACGTAGGAAGACGTGGCC
TaABCB1-DpromoterF	*GGGGACAAGTTTGTACAAAAAAGCAGGCT*ATCGTTCGGTATCCTGCTAG
TaABCB1-DpromoterR	*GGGGACCACTTTGTACAAGAAAGCTGGGT*GCATTGTTGGTGCCGGTAG
TaABCB1-3UTRF	*GGGGACAAGTTTGTACAAAAAAGCAGGCT*TGGTGGAGCAAGGGTCGCAC
TaABCB1-3UTRR	*GGGGACCACTTTGTACAAGAAAGCTGGGT*CGGATCATGGCATTGGCAGC
TaABCB1-19R	GTGTAGTAGATGCTGAGCAC
TaABCB1 D-27F	ATGTCTAGCGACCCTGAAGAG
TaABCB1 D-27R	CCAGACGCATTGTTGGTGCC
TaABCB1 A-29F	TCAACGCCGACGCTGTCATG
TaABCB1 A-29R	ACGAACGACTGCACGACTC
TaABCB1 B-30F	CAGTCCCCCACACACACTTG
TaABCB1 B-30R	GCTCTGCTGCTTCTGGCTCC
Act 247F	TGTGGATCTCCAAGGCGGAGTACG
HG8RBF	GTCAGGCTTTCCGACGTTTG
HG8C35SR	GGAATTAATTCGTCGAGCGGC
NPT IIF	GAGGCTATTCGGCTATGACTG
NPT IIR	ATCGGGAGCGGCGATACCGTA
NosF	ATGGCAATTACCTTATCCGCAACTTCTTTA
P27-5F	GGGATGACGCACAATCC
P27-3R	GAGCTACACATGCTCAGG
Act 247R	GCAGGCAAGCACCATGCAAGG
TaABCB1-hairpinF	GGCCGCATCATCGACCACACGCCAGGCGTCACCAAGGAAGGCGTGGATTCA<br>AGAGATCCACGCCTTCCTTGGTGACGCCTGGCGTGTGGTCGATGATTTAAT
TaABCB1-hairpinR	CGTAGTAGCTGGTGTGCGGTCCGCAGTGGTTCCTTCCGCACCTAAGTTCTCT<br>AGGTGCGGAAGGAACCACTGCGGACCGCACACCAGCTACTAAAT
TaABCB1- oligo	CGCGGCCGCGGCGAGCGCGTAGCCCCACTCGGGCGAGTTCATCTTGGCCA<br>GCCGCCAGAAGGAGCTGGCCTGCGCACGGAACGCGAGCTTCTCCATTCCCA<br>TGCCCATGCCCATCCGGTGGGCGGCCGGGTCATGTATGACGGACAGGCTGA<br>AGTCGGCGGTGGAGAAGTCGGAGAGGCGGCGGGAGTAGGGCGACCTTTAAT<br>TAACACCACACCAACGGCGGGCTGGCCATCGCCACCATGTTTTCCGTCATGA<br>TCGGCGGACTCGCTCTTGGGCAGTCGGCGCCGAGCATGGCGGCGTTTGCCA<br>AGGCGAGGGTTGCCGCCGCGAAGATCTTCCGTATCATCGACCACACGCCAG<br>GCGTCACCAAGGAAGGCGTGGAGCTGGAGTCGGTGACCGGGCGGCTGGAG<br>CTGAGGAACGTGGAGTTCGCGGCCGC

Table S2: *Cis*-regulatory elements and copy number identified in the promoter region of each of the homoeologous copies of *TaABCB1.*

Element	Motif	Function	A	B	D
**A-box**	CCGTCC	cis-acting regulatory element	+(2)	+(2)	+(1)
**3-AF3 binding site**	CACTATCTAAC	part of a conserved DNA module array (CMA3)	+(1)	-	-
**AAGAA-motif**	GAAAGAA		+(1)	+(2)	+(3)
	gGTAAAGAAA		+(2)		
**ABRE**	ACGTGGC	cis-acting element involved in the abscisic acid responsiveness	+(1)	-	-
**AC-II**	(C/T)T(T/C)(C/T)(A/C)(A/C)C(A/C)A(A/C)C(C/A)(C/A)C	xylem specific expression	+(1)	+(1)	-
**AE-box**	AGAAACAT	part of a module for light response	+(1)	+(1)	+(1)
**ARE**	TGGTTT	cis-acting regulatory element essential for the anaerobic induction	-	+(1)	-
**ATCT-motif**	AATCTAATCC	part of a conserved DNA module involved in light responsiveness	+(1)	+(1)	+(1)
**Box 4**	ATTAAT	part of a conserved DNA module involved in light responsiveness	-	-	+(1)
**CAAT-box**	CAAT	common cis-acting element in promoter and enhancer regions	+(11)	+(9)	+(24)
	CAAAT				
	CAAAT				
	CAAT				
	CCAAT				
	CAATT				
	TGCCAAC				
	gGCAAT				
	CAAT				
	CAAT				
	CAAAT				
	CCAAT				
	CAAAT				
	CAAT				
	CAAAT				

	CAAT				
	CAAAT				
	CAAT				
	CAAAT				
	CAAAT				
	CAAAT				
	CAAAT				
	CAAT				
**CCGTCC-box**	CCGTCC	cis-acting regulatory element related to meristem specific activation	+(2)	+(2)	+(1)
**CGTCA**	CGTCA	cis-acting regulatory element involved in the MeJA-responsiveness	+(1)	-	-
**G-Box**	CACGTT	cis-acting regulatory element involved in light responsiveness	+(4)	+(1)	+(3)
	CACGTT				
	CACGAC				
**GA-motif**	AAGGAAGA	part of a light responsive element	+(1)	-	-
**GAG-motif**	AGAGAGT	part of a light responsive element	+(1)	+(1)	+(2)
	AGAGATG				
**GARE-motif**	AAACAGA	gibberellin-responsive element	+(1)	+(1)	+(1)
**GATA-motif**	GATAGGA	part of a light responsive element	+(4)	+(1)	+(2)
	GATAGGG				
**GC-motif**	CCCCCG	enhancer-like element involved in anoxic specific inducibility	+(1)	-	-
**GCN4 motif**	TGAGTCA	cis-regulatory element involved in endosperm expression	+(1)	+(1)	-
**I-box**	GATAGGG	part of a light responsive element	+(1)	-	+(1)
**MBS**	CGGTCA	MYB Binding Site	+(2)	-	-
**LTR**	CCGAAA	cis-acting element involved in low-temperature responsiveness	-	-	+(1)
**MNF1**	GTGCCC(A/T)(A/T)	light responsive element	+(1)	+(1)	+(1)
**MRE**	AACCTAA	MYB binding site involved in light responsiveness	-	-	+(1)
**Skn-1_motif**	GTCAT	cis-acting regulatory element required for endosperm expression	+(2)	+(1)	+(3)

**Sp1**	CC(G/A)CCC	light responsive element	+(19)	+(3)	+(4)
**TATA-box**	TTTAAAAA	core promoter element around -30 of transcription start	+(10)	+(28)	+(16)
	TTTTA				
	TATA				
	TATAAAT				
	TTTTA				
	taTATAAAtc				
	TATACA				
	TATA				
	TTTTA				
	TACAAAA				
	TATA				
	TATAA				
	ATATAT				
	ATATAA				
	TATA				
**TATCCAT/C-motif**	TATCCAT	cis-acting regulatory element; associated with G-box like motif; involved in sugar repression responsiveness	+(1)	-	+(1)
**TC-rich repeats**	ATTTTCTTCA	cis-acting element involved in defense and stress responsiveness	+(2)	-	+(1)
	ATTTTCTCCA				
**TGACG-motif**	TGACG	cis-acting regulatory element involved in the MeJA-responsiveness	+(1)	-	-
**ASF1MOTIFCAMV**	TGACG	transcriptional activation of several genes by auxin and/or salicylic acid	+(2)	-	-
**SURECOREATSULTR11**	GAGAC	Core of sulfur-responsive element (SURE), SURE contains auxin response factor (ARF) binding sequence	+(3)	+(3)	+(1)
**Circadian**	CAANNNNATC	cis-acting regulatory element involved in circadian control	+(2)	+(1)	+(2)
